# From Microbes to Medicine: Targeting Metalloprotein Pathways for Innovative Antibacterial Strategies

**DOI:** 10.3390/ijms27020737

**Published:** 2026-01-11

**Authors:** Sumaya Sameer Alshatari, Malgorzata Ziarno

**Affiliations:** 1 Independent Researcher, 00-132 Warsaw, Poland; 2 Department of Food Technology and Assessment, Institute of Food Science, Warsaw University of Life Sciences—SGGW (WULS—SGGW), Nowoursynowska 159c St., 02-776 Warsaw, Poland

**Keywords:** metalloproteins, antimicrobial resistance, metal homeostasis, siderophores, drug development, translational strategies

## Abstract

Antibiotic resistance is an escalating global health problem that calls for new types of treatments beyond standard antibiotics. This review examines how targeting bacterial metalloproteins, especially those involved in siderophore-driven iron uptake and manganese-based oxidative defense, could lead to more selective antibacterial drugs that are less toxic to humans. Recent research shows that metals and metal-containing compounds can act as antimicrobials, but many of their biological roles are still not well understood. By synthesizing current evidence, this article critically evaluates translational strategies targeting bacterial metalloproteins. These include siderophore–antibiotic conjugates, metal trafficking inhibitors, and catalytic metallodrugs. The review suggests that therapies using receptor-mediated uptake and guided by genomic data deserve priority in clinical development. The review also highlights unresolved challenges in selectivity, toxicity, and resistance mechanisms, offering a roadmap for future research. This review integrates evidence from multiple databases to provide a comprehensive framework for targeting bacterial metalloproteins, combining narrative synthesis with systematic methodology.

## 1. Introduction

Bacterial infections result from a combination of biochemical pathways, structural changes, and interactions with the host that together support survival and virulence [1]. Among the systems that contribute most significantly to these processes are metalloproteins, which depend on essential metal ions such as zinc, iron, and manganese to support catalytic activity, redox balance, nutrient acquisition, and regulatory functions [2]. These metal-dependent pathways are essential for bacteria but often differ from those in humans, making them promising targets for selective drug design [3].

The accelerating global burden of antimicrobial resistance (AMR) highlights the limitations of traditional antibiotics, which primarily target cell wall synthesis, protein synthesis, or nucleic acid replication [4]. Although these agents have been highly effective, the rapid evolution of resistance mechanisms has diminished their long-term utility and underscores the need for innovative strategies that exploit alternative aspects of bacterial biology [5]. Metalloproteins and their associated metal-homeostasis networks offer such an opportunity. Their central roles in oxidative stress defense, metal acquisition, and enzymatic regulation position them as promising yet underexplored targets for next-generation antibacterial development [6].

This review synthesizes current knowledge on the functional importance of bacterial metalloproteins and examines how mechanistic insights can inform the design of novel therapeutic strategies. Particular emphasis is placed on siderophore-based delivery systems, inhibitors of metal trafficking and metalloenzymes, and emerging catalytic metallodrugs, all of which leverage the unique chemical properties of metal-dependent pathways to achieve selectivity and potency [7]. Figures and tables throughout the manuscript summarize the detailed mechanisms, structural features, and therapeutic modalities discussed, providing a visual framework that complements the conceptual overview presented here.

This review combines findings from structural biology, metal chemistry, and translational research to show the therapeutic potential of targeting bacterial metalloproteins and to outline the main challenges that affect drug design. Understanding these mechanisms is key to developing precise antibacterial drugs that can overcome multidrug resistance [8].

Collectively, these perspectives underscore the importance of metalloprotein systems as both biological determinants of virulence and promising therapeutic entry points. Following this conceptual framework, we next examine the molecular mechanisms governing bacterial metal homeostasis, specifically how pathogens regulate metal uptake, trafficking, and detoxification [9]. These mechanistic insights provide the foundation for evaluating metalloprotein-targeted antibacterial strategies in later sections.

## 2. Search Strategy and Evidence Selection

The literature underlying this review was identified through structured searches of PubMed, Scopus, and Web of Science, supplemented by manual screening of reference lists from relevant articles and reviews. The search covered publications from January 2005 to Dec antibacterial agents, including small molecules, biologics, and catalytic metallodrugs. Experimental formats included biochemical and biophysical studies, structural biology (X-ray crystallography, NMR, cryo-EM), microbiological assays, in vivo infection models, and early translational or pharmacological evaluations. Reviews were used primarily to contextualize established concepts and to identify additional primary literature.

Exclusion criteria were defined to preserve mechanistic depth and translational relevance. Non-peer-reviewed sources, conference abstracts without accompanying full articles, editorials, opinion pieces, and purely descriptive reports that lacked mechanistic or structural data were excluded. Studies focusing exclusively on eukaryotic or host metalloproteins without a clear bacterial component were also omitted. When multiple publications reported overlapping data from the same research group, preference was given to the most recent or comprehensive study. Titles and abstracts were initially screened for relevance, followed by full-text evaluation of selected articles. This structured yet narrative approach was chosen to balance breadth of coverage with mechanistic resolution, ensuring that the examples highlighted in the figures and tables are both representative of the field and informative for understanding the druggability of bacterial metalloprotein systems.

## 3. Mechanisms of Metalloprotein Targeting in Bacterial Pathogenesis

**Figure 1:**
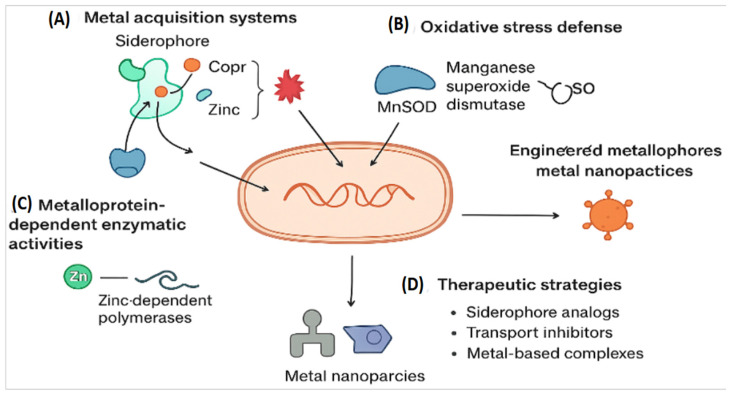
Mechanistic pathways of metalloprotein-mediated bacterial pathogenesis. (**A**) Copper resistance (Copr) operons and zinc transport systems facilitate detoxification and metal homeostasis under host-imposed stress. (**B**) Oxidative stress defense mediated by manganese- and iron-dependent superoxide dismutases (SODs), which neutralize reactive oxygen species (ROS). (**C**) Siderophore-mediated iron acquisition supports bacterial metabolism and virulence under metal-limited conditions. (**D**) Zinc-dependent polymerases contribute to DNA replication and repair, regulated by metal-sensor proteins and transcriptional reprogramming. These mechanisms illustrate how metalloproteins enable bacterial survival and adaptation in response to host-driven metal sequestration and intoxication.

Metalloproteins are essential for bacterial survival and virulence. Their role in infection can be grouped into four main mechanisms (Figure 1):

### 3.1. Oxidative Stress Response

Bacterial superoxide dismutases (SODs) are enzymes that neutralize superoxide radicals using metal-based redox reactions. Their activity depends on how metal ions, usually Fe^2+^/Fe^3+^ or Mn^2+^/Mn^3+^, are precisely arranged in the enzyme’s active site [10].

In *Staphylococcus aureus*, the dual-metal SOD (SodA) can incorporate either Fe or Mn, enabling functional flexibility under fluctuating host-imposed metal availability. The Mn-bound form adopts a five-coordinate distorted trigonal bipyramidal geometry [10]. The metal ion is ligated by His30, His81, Asp167, and His171. This coordination enables alternating oxidation states (Mn^2+^ ↔ Mn^3+^), allowing stepwise dismutation of superoxide into H_2_O_2_ and O_2_ [11]. This ability to switch metals helps bacteria survive during host-imposed metal limitation, such as calprotectin-driven restriction of Mn^2+^ and gFe^2+^. It is a major molecular adaptation that allows them to withstand oxidative stress [12].

### 3.2. Enzymatic Activity

Zinc-dependent enzymes are crucial for bacterial DNA replication, repair, and gene regulation [2]. Zn^2+^ typically functions as a structural or catalytic cofactor, coordinated in tetrahedral geometry by histidine, cysteine, or aspartate residues. Structural studies have shown that zinc uptake regulators (Zur family proteins) require Zn^2+^ binding at three distinct regulatory sites designated M-site, D-site, and C-site, to undergo conformational activation [9]. These sites exhibit unique coordination chemistries: the high-affinity M-site often involves cysteine Cys_2_His_2_ motifs, whereas the D-site incorporates mixed N/O ligands. Zn^2+^ binding induces allosteric rearrangements that stabilize DNA-binding helices, enabling precise transcriptional control of zinc homeostasis genes [13]. When this metal coordination is disturbed, the enzymes misfold and lose activity, showing how vital zinc is for maintaining bacterial DNA processes [14].

### 3.3. Metal Acquisition Systems

Bacteria use specific metal uptake systems to survive when the host limits access to essential nutrients [2]. These systems rely on metallophores, small, high-affinity chelators, that bind metal ions through defined coordination motifs [15]. *Acinetobacter baumannii* synthesizes multiple siderophores, including acinetobactin, baumannoferrin, and fimsbactin, each exhibiting distinct ferric-iron (Fe^3+^)-binding geometries [16]. Acinetobactin, the siderophore essential for virulence, coordinates Fe^3+^ through a catecholate–hydroxamate ligand set, forming an octahedral Fe^3+^ complex with sub-nanomolar affinity [7]. The Fe^3+^–acinetobactin complex is recognized by the TonB-dependent (TonB-dep.) transporter BauA, whose binding pocket is precisely shaped to accommodate the siderophore’s chelation geometry [16]. This molecular specificity explains why acinetobactin, despite the presence of other siderophores, is uniquely required for infection [16]. These systems show how bacteria have evolved highly refined ways to use metal–ligand chemistry for nutrient uptake [17].

### 3.4. Host–Pathogen Metal Competition

During infection, bacteria must navigate a chemically dynamic environment shaped by Host-driven metal sequestration and metal intoxication shape infection outcomes. Host proteins like calprotectin and lactoferrin block bacteria from getting manganese, zinc, and iron, while macrophages attack them with toxic levels of copper and zinc inside phagolysosomes [18]. In response, pathogens activate metal-responsive regulons that fine-tune metal uptake, efflux, and intracellular trafficking. Cupriavidus metallidurans exemplifies this adaptation through its integrated zinc-uptake (Zur) regulon, which coordinates multiple metal-binding proteins and transporters [19]. These systems rely on metal-sensor proteins with defined coordination motifs, often cysteine_2_-histidine_2_ (Cys_2_His_2_) or histidine_3_-aspartate (His_3_Asp), that detect subtle changes in intracellular Zn^2+^ levels [20]. Zn^2+^ binding induces conformational changes that modulate deoxyribonucleic-acid (DNA)-binding affinity, enabling rapid transcriptional reprogramming [21]. This connection between metal binding, protein structure, and gene regulation shows how complex bacterial survival strategies are. Such complexity becomes particularly evident under host-imposed metal limitation [2].

**Figure 2:**
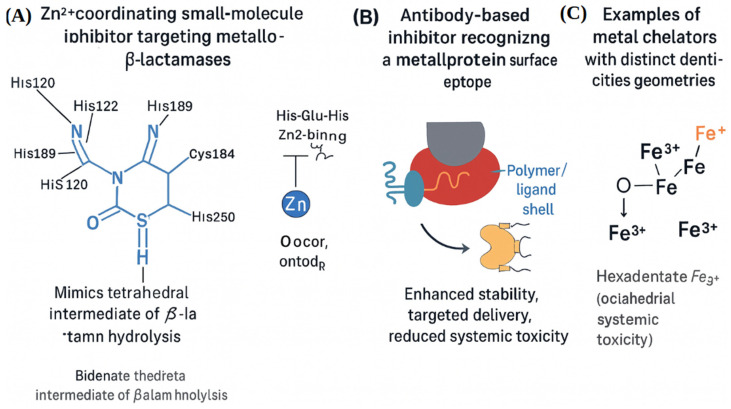
Structural diversity of antimicrobial agents targeting bacterial metalloproteins. (**A**) A Zn^2+^-coordinating small-molecule inhibitor mimicking the tetrahedral intermediate of β-lactam hydrolysis. The scaffold binds the dinuclear Zn^2+^ catalytic center of metallo-β-lactamases via bidentate O,O coordination, interacting with key active-site residues (His120, His122, His189, Cys184, His250). (**B**) A monoclonal antibody recognizing a surface-exposed metal-binding epitope on a bacterial metalloprotein. The antibody targets a His–Glu–His Zn^2+^ coordination motif, enabling selective neutralization of metal-dependent enzymatic activity. (**C**) A PEGylated nanoparticle with core–shell architecture and functionalized ligand shell. The nanoparticle delivers metal-targeting cargo, such as siderophore mimics or antibiotics, through bacterial porins or TonB-dependent receptors, enhancing stability and reducing systemic toxicity.

These regulatory mechanisms collectively govern bacterial survival, virulence, and resistance under host-imposed stress. The core mechanistic pathways by which bacterial metalloproteins contribute to survival and virulence under host-imposed stress are shown (Figure 1). Bacterial survival strategies include metal acquisition systems and oxidative stress defense mechanisms such as MnSOD, which operate together to maintain metal homeostasis under host-imposed stress (Figure 1). (Panel A) [15] and oxidative stress defense mechanisms like MnSOD (Panel B) [22]. Zinc-dependent polymerases and their associated therapeutic strategies are depicted (Figure 1, Panels C and D) [2,7]. These examples show that metal coordination is central to bacterial survival, and that it can be used to design precise antibacterial treatments. The molecular and nanostructural diversity of therapeutic strategies designed to disrupt metalloprotein activity is shown (Figure 2). A Zn^2+^-coordinating small-molecule inhibitor that mimics the tetrahedral intermediate of β-lactam hydrolysis is depicted (Figure 2A) [23]. This scaffold, exemplified by OP607 and α-aminophosphonate derivatives, transiently coordinates Zn^2+^ ions without permanently altering enzyme structure, enabling selective inhibition while preserving antibiotic efficacy in resistant strains. An antibody-based inhibitor targeting a surface-exposed metal-binding epitope is shown (Figure 2B) [24].

These biologics offer high specificity and can neutralize metalloprotein function without disrupting host metal homeostasis. A PEGylated nanoparticle system engineered for targeted delivery of metal-binding agents is illustrated (Figure 2C) [24]. The core–shell architecture and functionalized ligand shell enable receptor-mediated uptake through bacterial porins or TonB-dependent transporters, improving pharmacokinetics and reducing off-target toxicity [24].

These modalities, enzymatic inhibition, biological neutralization, and nanoparticle delivery, demonstrate how structural insights into metalloprotein coordination environments can guide the design of precision antibacterial agents. Their comparative evaluation reveals that siderophore-mediated uptake and dinuclear Zn^2+^ catalytic centers offer the most structurally tractable targets, while metal-switching enzymes and redundant detoxification systems pose greater challenges for inhibitor design.

Among these targets, New Delhi Metallo-β-lactamase-1 (NDM-1), a clinically significant zinc-dependent enzyme, has emerged as a key focus for inhibitor development due to its broad-spectrum β-lactam hydrolysis and its role in carbapenem resistance [23]. Recent studies have demonstrated that the reversible binding of small-molecule inhibitors, such as OP607 and α-aminophosphonate derivatives, to the zinc-coordinated active site of metallo-β-lactamases (MBLs) can restore antibiotic efficacy in resistant strains [24]. These compounds transiently coordinate the catalytic Zn^2+^ ions without permanently altering the enzyme structure, thereby enabling selective inhibition while minimizing off-target effects [25,26]. This reversible interaction competitively blocks access of β-lactam antibiotics to the active site, allowing these agents to retain antibacterial activity even in the presence of resistance determinants. For instance, NDM-1 hydrolyzes β-lactam antibiotics and serves as a critical resistance determinant in Klebsiella pneumoniae [23], while Mn-SOD protects pathogens such as *Staphylococcus aureus* from host-derived reactive oxygen species. Although mechanistically distinct, these targets share a reliance on metal cofactors, rendering them attractive candidates for selective inhibition strategies [22].

Although these systems are often discussed independently, their comparison within a unified mechanistic framework reveals shared molecular constraints that shape therapeutic tractability. Iron-siderophore uptake pathways exhibit highly rigid coordination geometries and receptor-specific recognition, making them attractive for pathogen-specific targeting [15]. In contrast, Mn-dependent oxidative stress enzymes such as MnSOD display metal-switching plasticity that complicates inhibitor design, as pathogens can substitute Fe or Mn depending on host-imposed metal restriction [22]. Zinc-dependent regulatory proteins occupy an intermediate position: their tetrahedral Zn^2+^ sites are structurally conserved, yet their regulatory networks exhibit redundancy that can buffer against single-target inhibition [24]. Copper detoxification systems, while mechanistically distinct, are often encoded by multi-gene operons with overlapping functions, reducing their vulnerability to small-molecule disruption [18]. Synthesizing these mechanistic differences highlights why some metalloprotein pathways, particularly siderophore-mediated uptake and dinuclear Zn^2+^ catalytic centers, offer clearer structural footholds for selective therapeutic intervention than others.

Notably, reversible inhibitors offer several advantages over irreversible covalent binders: they are less likely to induce compensatory mutations, can be structurally optimized for potency and spectrum, and often exhibit favorable pharmacokinetic profiles [24]. Building on these mechanistic insights, Key bacterial metalloproteins implicated in virulence, oxidative stress response, and nutrient acquisition are summarized (Table 1).

### 3.5. Unified Mechanistic Framework and Translational Progression

To clarify the conceptual contribution of this review, we propose a unified mechanistic framework that integrates structural, biochemical, and translational evidence across diverse bacterial metalloprotein systems. This framework compares metalloprotein targets based on metal-site rigidity, coordination chemistry, and pathogen-specific metal physiology, allowing their therapeutic tractability to be evaluated within a shared molecular context. Rigid metal-binding architectures, such as the octahedral Fe^3+^ coordination in siderophore receptors or the dinuclear Zn^2+^ centers of metallo-β-lactamases, offer well-defined structural footholds for selective inhibitor design. In contrast, metal-switching enzymes like Mn/Fe-dependent superoxide dismutases exhibit active-site plasticity that enables functional substitution under host-imposed metal restriction, reducing their vulnerability to small-molecule inhibition. Zinc-dependent regulatory proteins occupy an intermediate position: although their tetrahedral Zn^2+^ sites are structurally conserved, their regulatory networks often display redundancy that can buffer against single-target disruption [3]. Synthesizing these mechanistic differences highlights recurring molecular bottlenecks, such as metal-site remodeling, ligand-exchange dynamics, and host-driven metal competition, that constrain druggability across pathogens. This comparative perspective helps explain why certain metalloprotein pathways have advanced toward translational development, while others remain challenging targets despite their biological importance.

**Figure 3:**
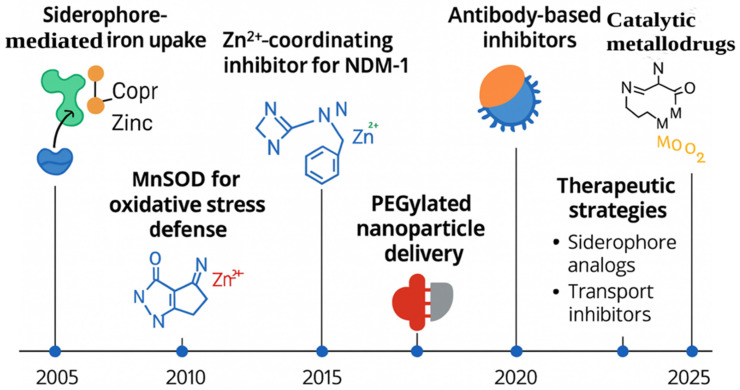
Development trajectory of antibacterial strategies targeting bacterial metalloproteins (2005–2025). This timeline highlights key milestones in the evolution of metalloprotein-targeted therapeutics. Early advances centered on siderophore-mediated iron uptake and oxidative stress enzymes such as MnSOD. Structural characterization of metallo-β-lactamases (e.g., NDM-1) enabled the rational design of Zn^2+^-coordinating inhibitors. More recent innovations introduced antibody-based inhibitors, PEGylated nanoparticle delivery systems, and catalytic metallodrugs. These milestones illustrate how insights into metal coordination, enzyme structure, and host–pathogen metal competition have shaped the development of mechanism-informed antibacterial strategies.

Building on this mechanistic foundation, A timeline of key discoveries and therapeutic innovations from 2005 to 2025 is provided (Figure 3). Each milestone illustrates how mechanistic insights have guided the evolution of antibacterial strategies targeting metalloproteins. Early advances centered on siderophore-mediated iron uptake and oxidative stress enzymes such as MnSOD. Structural characterization of metallo-β-lactamases, including NDM-1, enabled the rational design of Zn^2+^-coordinating inhibitors. More recent developments introduced antibody-based inhibitors, PEGylated nanoparticle delivery systems, and catalytic metallodrugs. This progression, from early siderophore systems to advanced catalytic metallodrugs, underscores the iterative nature of mechanism-informed therapeutic innovation and highlights how understanding metal coordination, enzyme structure, and host–pathogen metal competition continues to shape antibacterial strategy development.

## 4. Functional and Therapeutic Implications

Bacterial metalloproteins vary widely in structure but share core catalytic features that are essential for infection [2]. Many of them differ structurally from human proteins, which makes them promising drug targets with lower risk of host toxicity [13]. Crystallographic studies, including those by Šink et al. and Shirakawa et al., have provided detailed insight into key bacterial metalloenzymes involved in peptidoglycan synthesis [9]. These findings reveal weak points that can be targeted to design inhibitors that kill bacteria without harming host cells.

However, despite the identification of numerous metalloprotein targets, most clinically approved antibiotics still focus on traditional cellular processes, such as peptidoglycan synthesis, protein synthesis, and DNA replication. While these targets have historically provided effective treatments, the rise of resistance mechanisms has diminished their long-term efficacy. Antimicrobial resistance (AMR) causes around 1.27 million deaths each year and calls for a new approach that focuses on processes less likely to develop resistance—like metal homeostasis and metalloprotein function [14,21].

Despite progress, research still shows gaps and conflicting results that slow down translation into clinical use. In addition to metal chelation and enzyme inhibition, emerging studies also highlight complementary mechanisms of action, such as disruption of bacterial cell membrane integrity and inhibition of essential metabolic pathways, which may contribute to the antimicrobial effects of metal-based agents but remain underexplored in the current literature. Research consistently shows that siderophore systems bind iron with very high affinity, but studies disagree on how much transporter mutations reduce the effectiveness of siderophore–antibiotic conjugates in vivo. Although Zn-dependent enzymes are seen as good targets, structural studies show that many bacterial zinc-binding sites resemble those in human proteins, which raises concerns about off-target effects that often go unreported in preclinical work. Studies on MnSOD inhibition give mixed results. Knockout models confirm its role in virulence, but biochemical data suggest that disturbing redox balance could harm host cells [12]. These discrepancies underscore the importance of rigorous comparative studies and mechanistic validation instead of conclusions drawn from isolated experiments.

### From Mechanisms to Strategies: Integrating Metalloprotein Insights into Antibacterial Development

Understanding how bacterial metalloproteins work helps build the foundation for developing targeted antibacterial treatments. Because each bacterial species behaves differently, we need to move beyond broad-spectrum antibiotics and focus on drugs that target specific virulence mechanisms [8]. Insights from metalloprotein biology enable such precision by linking molecular function to therapeutic vulnerability.

Current antibiotics can be categorized based on their cellular targets, cell wall synthesis (β-lactams, glycopeptides), membrane disruption (polymyxins, lipopeptides), or interference with intracellular machinery (rifamycins, fluoroquinolones, tetracyclines) [8]. Yet many of these treatments fail to discriminate between pathogens and commensals, leading to microbiome disruption and secondary complications. Moreover, the overuse of these agents has accelerated the emergence of multidrug-resistant strains.

Current research focuses on developing new drugs that interfere with metalloprotein pathways, by binding essential metals, blocking metal uptake, or stopping metal-dependent enzymes. These strategies hold promises for overcoming resistance because they target fundamental physiological processes that are less amenable to genetic bypass. Furthermore, combining metalloprotein inhibitors with existing antibiotics could potentiate efficacy while minimizing dosage requirements and toxicity.

During the golden age of antibiotics (1940–1960), most drugs came from natural sources, but bacteria quickly developed resistance [28]. In contrast, metalloprotein-based approaches represent a new frontier in antimicrobial design, one that integrates structural biology, metal biochemistry, and drug discovery. Focusing on metal-dependent processes in bacteria could lead to safer and longer-lasting treatments in the post-antibiotic era.

When evaluated collectively, metalloprotein-targeting strategies reveal distinct mechanistic strengths and limitations that explain their uneven translational progress. Metal chelators can be powerful in the lab but often lack pathogen specificity and may also remove essential metals from the host, limiting their clinical use [29]. Siderophore-based approaches offer superior selectivity but remain vulnerable to transporter variability and the rapid evolution of bypass pathways. Catalytic metallodrugs show promise due to their mechanism-informed design; however, their stability and metal-release kinetics remain challenging to control in physiological environments. Inhibitors of metallo-β-lactamases such as New Delhi metallo-β-lactamase-1 (NDM-1) and Verona integron-encoded metallo-β-lactamase-2 (VIM-2) face additional challenges arising from active-site flexibility and metal-site heterogeneity, which reduce inhibitor binding consistency across clinical variants [8]. These comparisons show that developing effective therapies will require combining structural knowledge, evolutionary adaptability, and pathogen-specific metal biology, instead of relying on one type of approach.

## 5. Targeting Metal Homeostasis: Experimental Insights

Bacteria carefully regulate how they take up, use, and store essential metals like zinc, iron, copper, and manganese. These metals are vital for enzyme function, structural stability, and redox balance. Disturbing this balance could be a useful strategy for developing new antimicrobials, especially against multidrug-resistant bacteria.

Recent studies have clarified how bacteria regulate metal ions at the molecular level. For example, Colaço et al. characterized *Escherichia coli* ZinT, a periplasmic protein involved in resistance to toxic metals including cobalt, mercury, and cadmium [18]. Structural studies have identified metal-binding motifs that could help design inhibitors to block metal transport or interfere with metalloprotein formation.

Scientists have also studied in detail how metal availability affects bacterial virulence. Capdevila et al. demonstrated how pathogens maintain zinc metallostasis at the host–pathogen interface, using regulatory proteins and transport systems to evade host-imposed metal restriction [27]. These findings point to specific steps in bacterial metal uptake that could be targeted with new treatments.

The immune system also uses metal toxicity as a defense strategy against bacteria. Djoko et al. showed that high levels of copper and zinc in phagolysosomes help kill bacteria. This suggests that adjusting metal levels, or mimicking this effect with drugs, could be a useful antibacterial approach [6].

**Figure 4:**
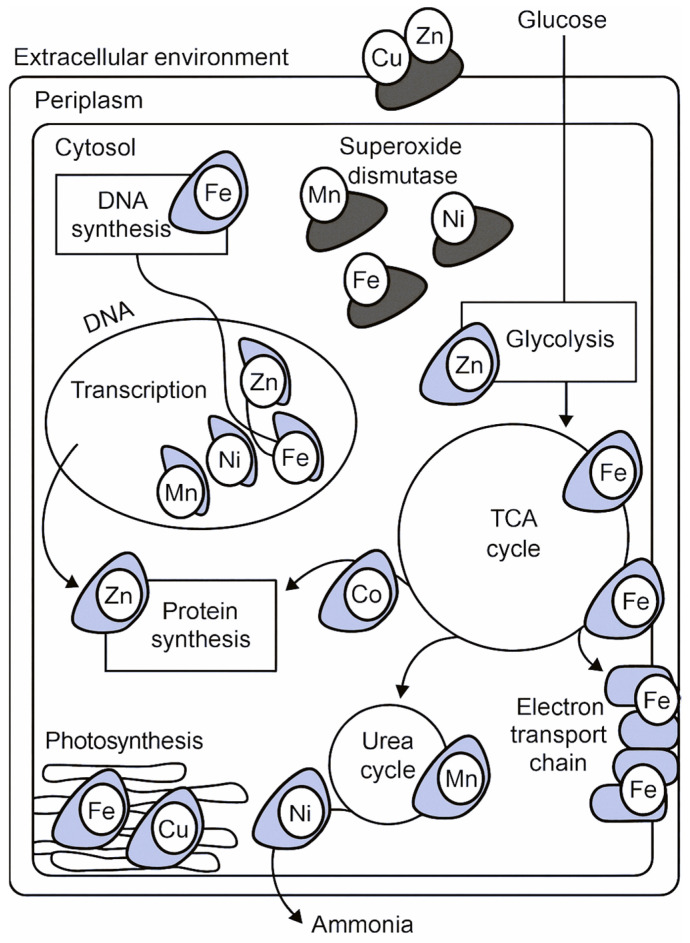
Cellular roles of metal ions in bacterial metabolism. The diagram shows how essential metals contribute to bacterial processes across compartments. Iron (Fe) supports DNA synthesis, the TCA cycle, electron transport, and photosynthesis. Zinc (Zn) is involved in transcription, protein synthesis, and glycolysis. Manganese (Mn), nickel (Ni), and cobalt (Co) facilitate superoxide dismutase activity, the urea cycle, and ammonia processing. Copper (Cu) and zinc (Zn) in the extracellular environment reflect host-imposed stress responses. Glucose uptake links nutrient acquisition to intracellular pathways.

Bacteria maintain metal balance using transporters, trafficking systems, chaperone proteins, and metal-storage molecules, as illustrated (Figure 4). Collectively, these systems keep redox balance and enzyme activity stable. When the host limits metal availability, bacteria respond with specialized uptake and detox mechanisms to survive.

Copper indirectly supports DNA synthesis by affecting redox balance and nucleotide metabolism. Copper-dependent enzymes regulate ribonucleotide reductase activity, a process essential for the production of deoxyribonucleotides. Dysregulated copper levels can impair DNA replication through oxidative stress and enzyme inhibition [6,27].

Manganese is a critical cofactor for arginase, the enzyme catalyzing the final step of the urea cycle, thereby regulating nitrogen disposal. Nickel is essential for bacterial urease, an enzyme that breaks down urea into ammonia and carbon dioxide, helping bacteria manage nitrogen and survive in the host [23].

Linking these mechanisms to the broader discussion helps readers see how biochemical pathways relate to treatment development. By clarifying how copper, manganese, and nickel contribute to DNA synthesis and nitrogen metabolism, the review provides a stronger rationale for targeting metalloproteins in the design of antibacterial agents [30].

The representative metalloprotein-targeting agents, including their mechanisms of action, delivery platforms, selectivity profiles, and clinical development stages, are listed (Table 1).

The main bacterial metalloprotein targets and their relevance to drug development are summarized (Table 2). Notably, *Mn-superoxide dismutase* in *S. aureus* has been identified as a validated virulence factor, with genetic knockout models confirming its role in oxidative stress resistance. Similarly, *iron-siderophore systems* in *E. coli* and *S. aureus* demonstrate pathogen-specific uptake mechanisms that can be exploited using siderophore analogs or chelators. While *copper transport proteins* in *P. aeruginosa* offer unique selectivity, their therapeutic targeting remains challenging due to isoform redundancy and host similarity [31]. These findings highlight the importance of designing drugs based on protein structure and tailoring them to each pathogen.

A cross-target comparison of the systems reveals recurring molecular themes that influence druggability across bacterial species, as summarized (Table 2). Targets with rigid metal coordination environments, such as Fe^3+^–siderophore receptors and dinuclear Zn^2+^ catalytic centers, tend to offer more predictable inhibitor binding modes and clearer structure–activity relationships. In contrast, pathways characterized by redundancy or metal-switching flexibility, including copper transport proteins and MnSOD, exhibit greater resilience to pharmacological disruption. Moreover, virulence-associated metalloproteins often display pathogen-specific structural features, whereas essential metabolic enzymes share higher homology with human counterparts, complicating selective targeting. These patterns explain why some strategies have advanced toward clinical evaluation while others remain confined to early-stage research, and they emphasize the importance of integrating comparative structural biology into target prioritization.

### 5.1. Mechanisms of Action

Metalloproteins incorporate essential metal cofactors into their active sites, where they mediate catalytic reactions through precisely defined coordination geometries and redox states. Their biochemical activity depends on the spatial arrangement of metal-binding residues, the electronic properties of the metal center, and the ability of the active site to stabilize reaction intermediates. Clinically relevant examples include New Delhi metallo-β-lactamase-1 (NDM-1), Verona integron-encoded metallo-β-lactamase-2 (VIM-2), and manganese-dependent superoxide dismutase (MnSOD), each illustrating distinct metal-dependent catalytic strategies [29].

Comparing these catalytic architectures highlights why inhibitor development has progressed unevenly across metalloprotein classes. Dinuclear Zn^2+^ enzymes, such as NDM-1 and VIM-2, provide well-defined coordination geometries that support rational inhibitor design; however, their flexible loop regions and variable Zn^2+^ occupancy introduce structural heterogeneity, which reduces inhibitor robustness across clinical variants. MnSOD, by contrast, possesses a highly conserved active site but relies on redox cycling and proton-coupled electron transfer, making selective inhibition challenging without perturbing host antioxidant pathways. These mechanistic distinctions illustrate that the success of metalloprotein-targeting strategies depends not only on identifying essential metal-dependent functions but also on understanding how active-site dynamics, metal coordination chemistry, and evolutionary plasticity shape inhibitor binding and resistance potential [34].

NDM-1 and VIM-2 hydrolyze β-lactam antibiotics through a dinuclear Zn^2+^ catalytic center. Structural studies reveal that NDM1 contains two zinc ions (Zn1 and Zn2) bridged by a hydroxide ion that acts as the catalytic nucleophile. Zn1 is coordinated by His120, His122, and His189, whereas Zn2 is coordinated by Asp124, Cys208, and His250. Together, this dinuclear Zn^2+^ center enables hydrolysis of the β-lactam ring. VIM-2 shares this general architecture but exhibits greater loop flexibility in the L3 and L10 regions, influencing substrate specificity and inhibitor susceptibility.

MnSOD, in contrast, relies on a single Mn^2+^ ion coordinated in a five-coordinate trigonal bipyramidal geometry by His26, His81, Asp167, His171, and a solvent ligand. Catalysis proceeds through a Mn^2+^/Mn^3+^ redox cycle, enabling the stepwise dismutation of superoxide radicals. The precise positioning of the metal center and hydrogen-bonding network stabilizes radical intermediates and ensures rapid turnover.

**Figure 5:**
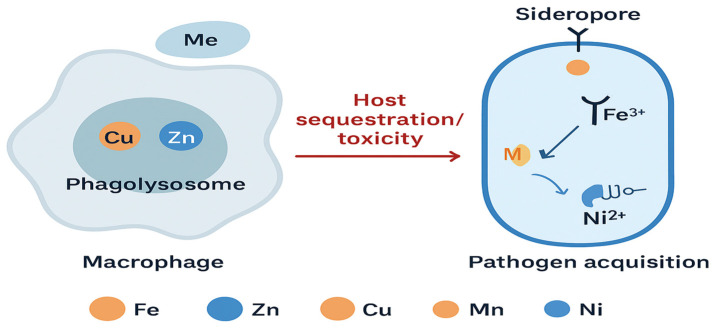
Molecular dynamics of host–pathogen competition for essential metal ions. This schematic illustrates the biochemical tug-of-war over transition metals during infection. Macrophage phagolysosomes mobilize toxic concentrations of copper (Cu^2+^) and zinc (Zn^2+^) to disrupt bacterial metalloproteins, while host proteins such as calprotectin sequester manganese (Mn^2+^) and Zn^2+^ to restrict microbial access. In response, pathogens activate high-affinity acquisition systems, including siderophores for ferric iron (Fe^3+^), Mn^2+^ transporters, and nickel (Ni^2+^)-dependent enzymes, to maintain metalloprotein function under metal-limited conditions. These opposing strategies directly influence catalytic efficiency, metal-site occupancy, and structural stability, shaping the molecular landscape for inhibitor design and therapeutic intervention.

These catalytic strategies operate within the broader context of host–pathogen metal competition, as illustrated (Figure 5). The figure depicts how macrophage phagolysosomes mobilize toxic concentrations of Cu^2+^ and Zn^2+^ to intoxicate invading bacteria, while host proteins such as calprotectin sequester Mn^2+^ and Zn^2+^ to restrict microbial access. In response, pathogens activate high-affinity acquisition systems, including siderophores for Fe^3+^, Mn^2+^ transporters, and Ni^2+^-dependent enzymes, to maintain essential metalloprotein function under metal-limited conditions. This biochemical tug-of-war directly shapes the catalytic efficiency, metal loading, and structural stability of bacterial metalloproteins.

Reversible inhibitors such as α-aminophosphonate derivatives exploit these vulnerabilities by mimicking the tetrahedral intermediate of β-lactam hydrolysis and coordinating Zn^2+^ through oxygen and nitrogen donors. By transiently occupying the dinuclear metal center, these inhibitors block substrate access without permanently modifying the enzyme, restoring antibiotic efficacy while minimizing off-target toxicity. Such mechanistic insights underscore the therapeutic potential of structure-guided targeting of metalloproteins [28].

### 5.2. Importance in Bacterial Survival

**Figure 6:**
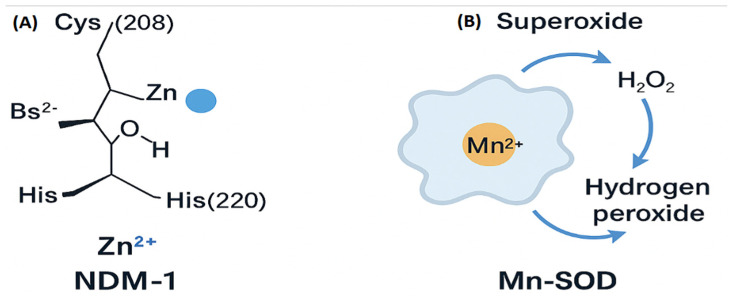
Molecular mechanisms and catalytic architectures of bacterial metalloproteins. (**A**) Active site structure of New Delhi metallo-β-lactamase-1 (NDM-1), showing dinuclear Zn^2+^ coordination with Cys208, His220, and a bridging sulfide. The Zn^2+^ cluster stabilizes a hydroxide nucleophile that initiates β-lactam hydrolysis via tetrahedral intermediate formation. This coordination geometry underpins inhibitor design strategies that mimic transition-state features and selectively bind the metal center. (**B**) Catalytic cycle of manganese-dependent superoxide dismutase (Mn-SOD), in which Mn^2+^ alternates between Mn^2+^ and Mn^3+^ oxidation states to convert superoxide radicals (O_2_^−^) into hydrogen peroxide (H_2_O_2_) and water. The trigonal bipyramidal geometry of the Mn^2+^ center, coordinated by conserved histidine and aspartate residues, enables redox cycling and radical stabilization. These structural features represent key vulnerabilities for the development of redox-active inhibitors.

Metals fulfill a dual function in bacterial physiology: they act as indispensable cofactors for enzymatic processes and simultaneously serve as stress-inducing agents deployed by host immunity. During infection, macrophage phagolysosomes release toxic concentrations of copper (Cu^2+^) and zinc (Zn^2+^) to impair bacterial viability, while host proteins, such as calprotectin, sequester manganese (Mn^2+^) and zinc, thereby limiting microbial access to these essential nutrients [6]. In countermeasure, pathogens activate high-affinity metal acquisition systems, including siderophores for iron (Fe^3+^), Mn^2+^ transporters, and Ni^2+^-dependent urease enzymes that facilitate nitrogen metabolism and enhance survival under hostile conditions [32,35]. The molecular architectures and catalytic strategies of two key bacterial metalloproteins are illustrated (Figure 6). The active site of New Delhi metallo-β-lactamase-1 (NDM-1), highlighting the dinuclear Zn^2+^ coordination environment that underpins β-lactam hydrolysis, is shown (Figure 6A). The zinc ion is ligated by residues such as Cys208 and His220, together with a bridging hydroxide and sulfide, forming a catalytic configuration that stabilizes the nucleophilic hydroxide responsible for β-lactam ring opening [23]. This structural arrangement exemplifies how precise metal coordination governs enzymatic activity and defines opportunities for transition-state-mimicking inhibitors. The catalytic cycle of manganese-dependent superoxide dismutase (Mn-SOD), in which a Mn^2+^ center alternates between Mn^2+^ and Mn^3+^ oxidation states to convert superoxide radicals into hydrogen peroxide, is depicted (Figure 6B). This redox-driven detoxification mechanism enables bacteria to withstand host-derived oxidative stress and maintain intracellular redox balance [29].

These panels demonstrate how metal coordination geometry and redox chemistry shape bacterial survival pathways, providing mechanistic entry points for therapeutic strategies aimed at disrupting microbial metal homeostasis.

### 5.3. Potential for Drug Development

Historically, metal-based compounds such as gold salts and arsenicals were used to treat infections and inflammatory diseases. Modern bioinorganic chemistry has revived interest in metal-containing antibiotics that disrupt bacterial metabolism through redox cycling, membrane destabilization, or enzymatic inhibition [28,29].

Two primary mechanisms define metal-based antibacterial action:

- Extracellular disruption: Charged species and ionic metals destabilize the bacterial envelope and proton motive force.
- Intracellular targeting: Metal pharmacophores penetrate cells and undergo organometallic transformations triggered by bacterial reductants.

For example, OP607, a nanoparticle-based iron chelator, has demonstrated biofilm inhibition with low toxicity [8,33]. Similarly, α-aminophosphonate inhibitors of NDM-1 selectively bind zinc at the β-lactamase active site, restoring antibiotic efficacy in resistant strains [14].

**Figure 7:**
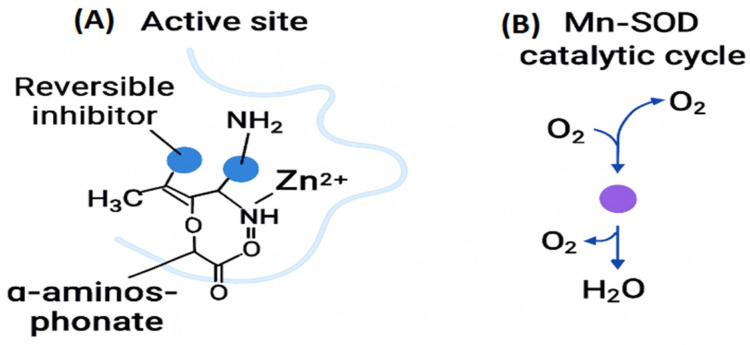
Catalytic architectures and inhibition strategies targeting bacterial metalloproteins. (**A**) Structural representation of an α-aminophosphonate inhibitor reversibly coordinating Zn_2+_ in the active site of New Delhi metallo-β-lactamase-1 (NDM-1). The phosphonate moiety forms bidentate O,O-donor interactions with the dinuclear Zn_2+_ cluster, displacing the catalytic hydroxide and mimicking the tetrahedral oxyanion intermediate of β-lactam hydrolysis. The amine group forms hydrogen bonds with His120 and His122, enabling selective targeting of metalloproteins. (**B**) Catalytic cycle of manganese superoxide dismutase (Mn-SOD), illustrating the dismutation of superoxide radicals (O_2_^−^) into molecular oxygen (O_2_) and hydrogen peroxide (H_2_O_2_), mediated by the redox-active manganese center.

Two complementary catalytic strategies relevant to metalloprotein targeting are illustrated (Figure 7). The reversible coordination of Zn^2+^ by an α-aminophosphonate inhibitor within the NDM-1 active site is shown (Figure 7A). The inhibitor engages its amino, carbonyl, and alkyl substituents to position the phosphonate group for bidentate interaction with the zinc ion, demonstrating how precise metal chelation can disrupt β-lactamase catalysis without permanently altering the enzyme [28]. The catalytic cycle of manganese-dependent superoxide dismutase (Mn-SOD), in which a Mn^2+^ center alternates between Mn^2+^ and Mn^3+^ oxidation states to convert superoxide radicals into molecular oxygen and hydrogen peroxide, is depicted (Figure 7B). This redox-driven detoxification pathway highlights the essential role of metal-dependent enzymes in protecting bacteria from oxidative stress [36].

Together, the two panels underscore how metal coordination geometry and catalytic chemistry can be strategically exploited to impair bacterial survival and guide the development of selective metalloprotein-targeted therapeutics.

## 6. Innovative Approaches to Target Metalloproteins: Toward Mechanism-Informed Antibacterial Design

This section focuses on how metal coordination, catalysis, and redox processes can be used to design more effective antibacterial agents. Instead of reviewing every metalloprotein system, we focus here on the molecular rules that determine how inhibitors interact with metal-dependent enzymes and receptors. This structure enables direct comparison across therapeutic modalities and highlights the mechanistic logic underlying successful antibacterial design [37].

Metalloproteins, which depend on metals like zinc, manganese, and copper, are valuable antibacterial targets because their metal-binding sites are highly conserved and essential for bacterial survival. The catalytic and structural roles of these metals create vulnerabilities that can be exploited through the disruption of coordination geometry, interference with redox cycling, or perturbation of metal trafficking pathways [35]. Recent advances in structural biology, cryo-EM, and modeling tools like AlphaFold make it possible to study these metal sites in detail and design inhibitors based on their actual structure rather than trial and error [38].

Within this mechanistic framework, we examine three therapeutic modalities, small-molecule inhibitors, biologics, and catalytic metallodrugs, each of which leverages distinct molecular strategies to impair metalloprotein function. By comparing their coordination preferences, binding modes, and mechanistic bottlenecks, this section provides a focused, molecularly grounded synthesis of emerging approaches to target bacterial metalloproteins [37].

Studies show that metalloprotein-targeting strategies differ greatly in selectivity, stability, and potential for clinical use, and each comes with its own limitations. Traditional metal chelators can bind many metals but lack specificity, often disrupting host proteins and performing poorly in living systems because of weak pharmacokinetics. In contrast, structure-based inhibitors like α-aminophosphonates or hydroxamates are more selective but can still be affected by resistance, for example, through mutations in the active site or changes in the metal-binding region. Biologic drugs are highly specific for surface metal-binding regions, but their large size limits how well they can reach tissues or work inside bacterial cells. Catalytic metallodrugs and metalloPROTACs bring new mechanisms, but their high reactivity and unstable metal centers raise concerns about side effects and stability inside cells [39]. These inconsistencies reveal a key challenge in translating these findings into clinical applications. Many inhibitors work well in lab conditions but lose effectiveness in living systems. In the body, metal levels, redox balance, and host immunity can change how metalloproteins fold and function [39]. Comparing these strategies shows that we need infection models that reflect real metal conditions and treatments that target more than one bacterial pathway, since bacteria can adapt easily [8].

### 6.1. Small Molecule Inhibitors: Beyond Chelation

Small-molecule inhibitors have come a long way from older chelators that simply removed metal ions and often interfered with essential host proteinss. Modern inhibitor design instead relies on precise, structure-guided engagement of catalytic metal centers, exploiting the unique coordination geometries, ligand preferences, and electronic properties that distinguish bacterial metalloproteins from their human counterparts [12]. This mechanistic refinement enables selective inhibition of bacterial enzymes while minimizing off-target toxicity.

**Figure 8:**
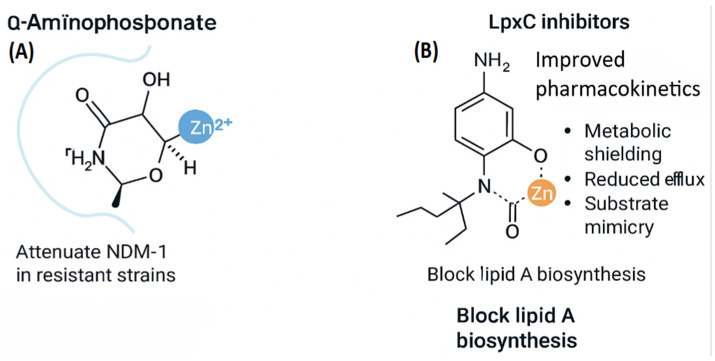
Molecular mechanisms of zinc-coordinated small-molecule inhibitors. (**A**) α-Aminophosphonate inhibitor targeting the dinuclear Zn^2+^ catalytic center of NDM-1. The phosphonate group forms bidentate O,O-donor coordination with Zn1 and Zn2, displacing the catalytic hydroxide and mimicking the tetrahedral oxyanion intermediate of β-lactam hydrolysis. Stabilizing interactions with His120, His122, and His189 anchor the inhibitor within the active site, blocking nucleophilic attack and restoring β-lactam efficacy. (**B**) Hydroxamate-based LpxC inhibitor chelating Zn^2+^ in a bidentate fashion. Hydrophobic substituents engage the substrate-binding tunnel, enhancing pharmacokinetics through metabolic shielding, reduced efflux susceptibility, and substrate mimicry. This design blocks lipid A biosynthesis, a critical step in Gram-negative outer membrane formation.

Modern inhibitor design focuses on binding directly to the catalytic metal center instead of just removing metals indiscriminately. *New Delhi metallo-β-lactamase-1 (NDM-1)* exemplifies this approach. The dinuclear Zn^2+^ cluster of NDM-1, in which Zn1 is coordinated by His120, His122, and His189 and Zn2 by Asp124, Cys208, and His250, is shown (Figure 8A). The two Zn^2+^ ions are bridged by a hydroxide that serves as the nucleophile during β-lactam hydrolysis. α-Aminophosphonate inhibitors exploit this architecture by forming bidentate O,O-donor interactions with the Zn^2+^ cluster, mimicking the tetrahedral oxyanion intermediate of the hydrolytic reaction [39]. The phosphonate moiety displaces the bridging hydroxide, while the amine group forms stabilizing hydrogen bonds with His120 and His122, anchoring the inhibitor within the active site. This transition-state mimicry effectively blocks β-lactam hydrolysis while preserving the native metal-binding geometry, thereby reducing selective pressure for resistance-conferring mutations [40].

Besides metallo-β-lactamases, LpxC inhibitors target other zinc-dependent enzymes that play a key role in lipid A biosynthesis. These inhibitors incorporate zinc-binding pharmacophores that coordinate the catalytic Zn^2+^ ion via bidentate O,O-donor groups, such as hydroxamates or phosphonates, as shown (Figure 8B) [12]. Structural optimization enhances pharmacokinetics through metabolic shielding, reduced efflux, and substrate mimicry, enabling selective inhibition of LpxC without disrupting host metalloproteins. By blocking lipid A synthesis, these compounds compromise outer membrane integrity and sensitize Gram-negative bacteria to immune clearance and antibiotic co-therapy [12].

Structure–activity relationship (SAR) analyses across metalloprotein families underscore the importance of ligand denticity, donor atom identity, and chelation geometry:

- Bidentate O,O-donors (e.g., phosphonates, hydroxamates) enhance Zn^2+^ affinity by stabilizing tetrahedral or trigonal bipyramidal geometries.
- Mixed N,O-donors can be tuned for Fe^3+^ or Mn^2+^ selectivity by modulating ligand field strength.
- Electron-donating substituents on aromatic scaffolds increase metal affinity by enhancing electron density at the donor atoms.
- Steric bulk near the metal-binding group reduces potency by hindering access to the catalytic pocket or distorting optimal chelation geometry.

LpxC inhibitors constitute a second major class of mechanism-informed metalloprotein inhibitors. These compounds target the zinc-dependent deacetylase LpxC, a key enzyme in lipid A biosynthesis, via a hydroxamate moiety that chelates Zn^2+^ in a bidentate manner [41]. Hydrophobic substituents extend into the enzyme’s substrate-binding tunnel, enhancing affinity through shape complementarity and van der Waals interactions. Modern LpxC inhibitor design incorporates several pharmacokinetic optimizations, including steric shielding of the hydroxamate to reduce metabolic degradation, polarity tuning to minimize efflux via RND transporters, and scaffold rigidification to decrease entropic penalties upon binding and enhance target specificity [8].

Overall, these advances show a move away from non-specific chelation toward structure-based inhibitor design, where the metal’s coordination and active-site geometry help create more selective and effective antibacterials. By integrating structural biology, metal-ligand theory, and SAR-based optimization, small-molecule inhibitors can effectively disrupt metalloprotein function and restore antibiotic efficacy against multidrug-resistant pathogens.

Even though α-aminophosphonate and hydroxamate inhibitors show strong effects in vitro, their reported activity varies a lot between studies. These differences often come from variations in metal levels, zinc binding, and enzyme flexibility, showing why we need more standardized, metal-aware assays that reflect real physiological conditions [8].

### 6.2. Monoclonal Antibodies and Biologics: Precision Targeting

Biologics offer unmatched specificity for surface-exposed metalloproteins, particularly those involved in host invasion and oxidative stress regulation. Engineered tissue inhibitors of metalloproteinases (TIMPs) and antibody–drug conjugates (ADCs) exemplify this modality [40]. However, their clinical deployment faces several biophysical and pharmacological challenges. Limited tissue penetration arises from the large molecular size of IgG antibodies (~150 kDa) and their restricted diffusion across epithelial and stromal barriers; glycosylation patterns and surface charge further impede movement through dense extracellular matrices. Binding-site barriers can also occur, where high-affinity interactions near vasculature sequester antibodies peripherally and prevent deeper penetration into infected tissues. Additionally, repeated administration in chronic infections increases the risk of immunogenicity, as recombinant proteins may elicit anti-drug antibodies that reduce efficacy or accelerate clearance. Recent advances in nanobody engineering and bispecific antibody formats address these limitations: nanobodies (~15 kDa) exhibit superior tissue penetration and reduced immunogenicity, while bispecific constructs enable simultaneous targeting of metalloproteins and immune effector pathways. Biologics directed against manganese superoxide dismutase (MnSOD) or copper-transport proteins can synergize with host nutritional immunity, enhancing pathogen clearance while minimizing selective pressure for resistance [36,37].

Even though biologics are highly specific, they often fail in deep-tissue infections because the relevant metal-binding sites are not always accessible in all bacterial states.

### 6.3. Catalytic Metallodrugs and Metallo-PROTACs: Mechanistic Innovation

Catalytic metallodrugs are a unique class of antibacterials that use the redox activity and flexibility of transition metals to disrupt key bacterial processes. Unlike stoichiometric inhibitors, catalytic metallodrugs undergo multiple turnover cycles, enabling sustained antibacterial activity at comparatively low concentrations. Their mechanisms derive from the intrinsic reactivity of metal centers such as Cu^2+^/Cu^+^, Ni^2+^/Ni^3+^, and Ru^2+^/Ru^3+^, which can participate in electron transfer, radical generation, and metal-center substitution reactions within the bacterial cytosol [35].

**Figure 9:**
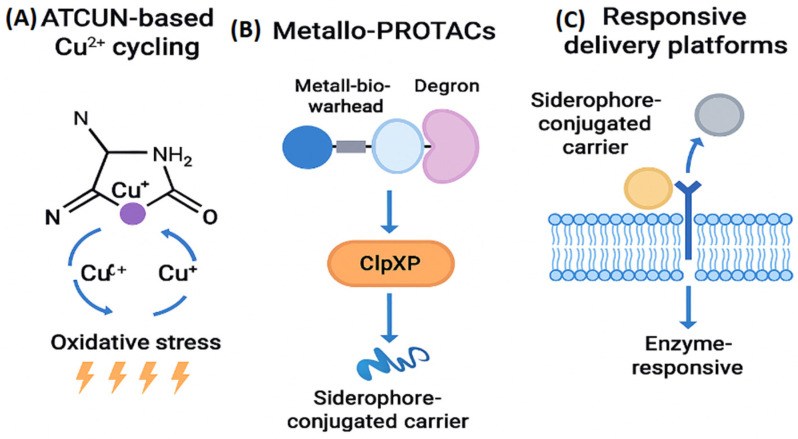
Metal-driven strategies for bacterial disruption. (**A**) ATCUN-based catalytic metallodrugs coordinate Cu^2+^ via N_3_O donor atoms and undergo Cu^2+^/Cu^+^ redox cycling, generating reactive oxygen species (ROS) that induce oxidative damage to bacterial DNA, lipids, and proteins. (**B**) Metallo-PROTACs combine a metal-binding warhead with a bacterial degron motif, recruiting ClpXP protease complexes to selectively degrade intracellular metalloproteins. The construction includes a siderophore-conjugated carrier for targeted uptake. (**C**) Enzyme-responsive delivery platforms use siderophore-conjugated carriers to transport metallodrugs across bacterial membranes. Drug release is triggered by pathogen-specific enzymatic activity, enhancing localization and minimizing off-target effects.

ATCUN-based metallodrugs (Amino-Terminal Cu^2+^/Ni^2+^-binding motifs) exemplify this catalytic paradigm. A conceptual overview of how metalloprotein inhibition disrupts bacterial physiology is provided (Figure 9A). This geometry stabilizes the Cu^2+^/Cu^+^ redox couple and enables Fenton-like cycling, generating reactive oxygen species (ROS) such as hydroxyl radicals (•OH). These ROS induce oxidative cleavage of DNA, lipid peroxidation, and protein carbonylation. Because bacterial cells possess weaker redox buffering systems and lower glutathione reserves than mammalian cells, ATCUN-mediated oxidative stress preferentially damages bacterial biomolecules while sparing host tissues [35].

A second mechanistic strategy involves metal-center substitution, in which catalytic metallodrugs exchange their metal ion with the native cofactor of a bacterial metalloprotein. For example, Fe-mimetic complexes can replace Fe^2+^ in ribonucleotide reductase or dehydratases, generating inactive or mis-metalated enzymes. This mechanism exploits the strict metal selectivity of bacterial enzymes and the vulnerability of pathogens to metal imbalance. Ligand-exchange reactions are facilitated by the labile coordination spheres of many catalytic complexes, which undergo rapid substitution under intracellular reducing conditions.

Metallo-PROTACs extend catalytic principles into the realm of targeted protein degradation. These chimeric molecules couple a metal-binding warhead, designed to engage the active site of a metalloprotein, with a degron motif recognized by bacterial proteases such as ClpXP or Lon (Figure 9B). Although bacteria lack ubiquitin-dependent degradation pathways, engineered degrons can recruit these ATP-dependent proteases to selectively degrade intracellular metalloproteins. The linker connecting the warhead and degron must balance metal-binding geometry, conformational flexibility, and bacterial permeability, ensuring that the chimera can traverse the cell envelope and maintain productive orientation within the protease complex.

A major challenge in metallo-PROTAC design is the lability of metal–ligand interactions, which can lead to premature dissociation or off-target reactivity. To address this, recent designs incorporate chelation-stabilized warheads, redox-inert metal centers, or prodrug strategies that activate the metal complex only after bacterial uptake. These refinements improve intracellular stability and reduce unintended interactions with host metalloproteins [39].

Responsive delivery platforms further enhance the precision of catalytic metallodrugs. Siderophore-conjugated carriers exploit bacterial iron-uptake pathways to transport metallodrugs across the outer membrane, while enzyme-responsive linkers release the active compound in the presence of pathogen-specific proteases (Figure 9C). These systems localize drug activity to infection sites, minimize systemic toxicity, and preserve host metal homeostasis.

Together, catalytic metallodrugs and metalloPROTACs show how metal chemistry and targeted degradation can be used to block essential bacterial pathways. Their multi-turnover activity, unique mechanisms, and compatibility with pathogen-specific delivery systems position them as promising candidates for next-generation antibacterial development.

Catalytic metallodrugs demonstrate strong mechanistic rationale, yet discrepancies between studies reflect challenges in controlling redox cycling and metal-center stability under physiological conditions.

### 6.4. Molecular Bottlenecks and Future Directions in Metalloprotein-Targeted Antibacterial Design

Progress in metalloprotein-targeted antibacterial development depends on integrating structural biology, bioinorganic chemistry, and infection-specific pharmacology to create mechanism-informed therapies. Although recent advances, such as α-aminophosphonate MBL inhibitors, LpxC inhibitors, nanobody formats, and catalytic metallodrugs, demonstrate translational promise, several fundamental challenges continue to limit progress. A comparative analysis of current strategies reveals that many promising approaches falter because they do not fully account for the molecular constraints imposed by metal availability, host immunity, and the structural dynamics of metalloproteins.

A major limitation across literature is the lack of standardized, physiologically relevant infection models. Many preclinical systems fail to reproduce host-imposed metal restriction or intoxication, spatial metal gradients in tissues, redox conditions generated during inflammation, or the biofilm-associated microenvironments that reshape metalloprotein expression and metal-uptake pathways. Because metalloprotein function is tightly conditioned by metal availability and immune pressure, these discrepancies reduce the predictive value of in vitro and in vivo studies. Establishing harmonized, metal-aware models, incorporating nutritional immunity factors such as calprotectin, realistic Zn/Mn gradients, copper-mediated toxicity, and infection-site pharmacokinetics, will be essential for improving reproducibility and identifying therapeutics with genuine translational potential [8].

Equally important is the need to address the redundancy and adaptability of bacterial metal-homeostasis networks. Comparative studies show that bacteria frequently evade single-inhibitor pressure by activating alternative siderophore systems, expressing paralogous metalloenzymes, rewiring oxidative-stress responses, or altering membrane permeability and metal flux. These molecular escape routes explain why several inhibitor classes show strong in vitro activity yet limited in vivo durability. Future antibacterial drugs should hit more than one target within metal-dependent pathways to make it harder for bacteria to adapt. Promising approaches include:

- dual-pathway inhibition (e.g., siderophore blockade + MnSOD inhibition)
- rational drug combinations informed by network topology
- bifunctional inhibitors coupling metal-binding pharmacophores with allosteric modulators
- catalytic metallodrugs or metallo-PROTACs capable of disabling multiple essential nodes

Together, model standardization and redundancy-aware multi-target strategies form a coherent roadmap for future innovation. Evaluating inhibitors within realistic microenvironmental constraints will sharpen mechanistic insight and translational accuracy, while multi-target agents will reduce evolutionary escape and increase therapeutic durability. As next-generation metalloprotein inhibitors continue to mature, integrating these principles will be crucial for achieving selective, resistance-resilient, and clinically actionable antibacterial therapies.

Within the unified framework presented here, these comparisons indicate that no single therapeutic modality overcomes all molecular bottlenecks. This underscores the need for integrated, multi-mechanism approaches and highlights the conceptual contribution of this review: a unified framework that identifies shared vulnerabilities across metalloprotein systems and clarifies which molecular features most strongly influence therapeutic tractability.

## 7. Critical Appraisal and Innovation Pathways

Targeting bacterial metalloproteins marks a clear change in how we design antibiotics. Instead of broadly blocking essential bacterial processes, this approach focuses on disrupting metal use, redox balance, and enzyme-based detox systems that bacteria depend on [38]. Because bacterial and human metalloproteins differ, targeting these metal-dependent systems could lead to narrow-spectrum drugs that spare beneficial microbes and reduce side effects [39]. Still, case studies on siderophore–antibiotic conjugates, metallo-β-lactamase inhibitors, MnSOD inhibitors, and catalytic metallodrugs show clear technical and clinical challenges that need to be solved before this approach can fully deliver.

### 7.1. Isoform Selectivity and Structural Precision

Selectivity is still one of the biggest hurdles, since many metal-binding sites look similar in bacteria and humans. Studies show that it’s possible to reach isoform-level specificity, but it requires very detailed structural data on active sites and metal coordination. For example, NDM-1 inhibitors such as α-aminophosphonates exploit the conserved dinuclear Zn^2+^ center and surrounding residues (His120, His122, His189, Asp124, Cys208) to achieve selective coordination and transition-state mimicry, restoring β-lactam activity without indiscriminate Zn^2+^ chelation [11,12,14]. In contrast, broad Zn-chelating agents, while potent, show poor isoform discrimination and systemic toxicity, highlighting the risk of targeting metal ions without structural anchoring [5,25].

New tools like cryo-EM and AlphaFold now let us examine metalloproteins at the isoform level and uncover hidden pockets or pathogen-specific motifs that can’t be predicted from sequence alone [41,42]. These tools have already improved docking accuracy for Zn-dependent enzymes and can be extended to siderophore receptors, MnSOD variants, and copper transporters [8,28]. A concrete innovation pathway is therefore to prioritize targets for which high-resolution structures (experimental or AlphaFold-supported) are available and to use these models to design non-chelating pocket binders that preserve native metal coordination while engaging isoform-specific cavities. The successful differentiation between NDM-1 and VIM-2 inhibitor profiles [12,43] exemplifies how structure-guided discrimination between closely related metalloprotein isoforms can be achieved.

### 7.2. Delivery Platform Optimization

Even the most selective inhibitors will fail if they cannot reach the infection site in effective concentrations. Siderophore–antibiotic conjugates show how receptor-based delivery can help drugs cross the tough outer membranes of Gram-negative bacteria. Cefiderocol leverages catecholate-based Fe^3+^ chelation to hijack TonB-dependent transporters such as FepA and CirA, enabling efficient uptake into the periplasm and potent activity against carbapenem-resistant strains [38,40]. This “Trojan horse” concept is mirrored by experimental siderophore–drug conjugates that exploit pathogen-specific siderophore receptors identified in *Acinetobacter baumannii* and *Pseudomonas aeruginosa* [44,45].

Nanoparticle systems offer another option. PEG-coated or siderophore-linked particles can carry metal-binding drugs straight to infection sites, improving stability and lowering systemic exposure [37,39]. For instance, OP607 demonstrates that nanoparticle-integrated iron chelation can inhibit biofilms with low toxicity [32]. These examples argue for an innovation pathway that systematically matches each metalloprotein target class with an appropriate delivery modality: siderophore-based uptake for outer-membrane-restricted Gram-negative targets; antibody or nanobody formats for surface-exposed metalloproteins; and nanoparticle or prodrug systems for intracellular enzymes such as NDM-1, VIM-2, or MnSOD.

### 7.3. Multi-Target Resistance Mitigation

Because bacterial metal networks are flexible and redundant, drugs that hit only one target rarely work for long. Case studies highlight this limitation clearly. Siderophore–antibiotic conjugates are vulnerable to transporter mutations and pathway bypass, while MnSOD-directed interventions face compensatory activation of alternative oxidative-stress pathways and metal switching (Mn^2+^ ↔ Fe^2+^) [12,17]. Similarly, gallium-based iron mimetics disrupt Fe^3+^-dependent pathways but show limited spectrum and host toxicity constraints at therapeutic doses [46,47].

To limit resistance, treatments should act on multiple targets within the same network. One concrete approach is rational drug combinations that simultaneously block siderophore-mediated iron uptake and neutralize metallo-β-lactamase activity, thereby restricting both nutrient acquisition and enzymatic resistance. Another is dual-pathway inhibition, such as combining MnSOD inhibition with compounds that interfere with metal import (e.g., Mn^2+^ transporters) or redox buffering, forcing bacteria into unsustainable oxidative stress [48,49]. Comparative genomics tools such as gSpreadComp and AMR-focused sequencing platforms can help prioritize metalloprotein targets that are both conserved and central within virulence networks, reducing the likelihood of escape through alternative pathways [45,50]. These examples move the concept of “multi-targeting” from an abstract recommendation to specific, testable combinations anchored in case-study evidence.

### 7.4. Interdisciplinary Integration for Clinical Translation

The studies reviewed here show that real progress happens only when structural biology, chemistry, microbiology, and systems analysis are combined from the start. For example, the optimization of α-aminophosphonate scaffolds required iterative cycles of crystallography, SAR analysis, and microbiological validation to align metal-site binding with whole-cell activity [11,12]. Likewise, cefiderocol’s advancement into clinical use relied not only on siderophore chemistry but also on pharmacokinetic studies in patients with renal impairment and detailed surveillance of resistance evolution [51,52].

Systems biology and metagenomic approaches add a further layer, enabling prediction of how metalloprotein-targeted agents will reshape microbial communities and resistance networks [53,54]. For instance, the impact of iron-targeting strategies on gut microbiota composition and resistome dynamics can now be tracked using shotgun sequencing and mobile-element profiling [43,55]. This supports more informed risk–benefit assessments when deploying siderophore-based drugs or metal chelators.

In practice, innovation depends on four things: choosing targets with known structure, matching each target with the right delivery system, designing combinations that work across pathways, and testing pharmacokinetics and safety early in development [29,42].

Of the strategies reviewed, receptor-mediated delivery and non-chelating pocket binders look most promising. They are specific, work well with existing antibiotics, and are supported by solid structural data [51,52]. Biologics, including monoclonal antibodies and engineered TIMPs, offer exceptional selectivity but are limited by cost, immunogenicity, and penetration into deep-seated infections [11,16]. Catalytic metallodrugs and metalloPROTACs, such as ATCUN or platinum complexes, are exciting but still need careful testing for metal stability, side reactions, and behavior in living systems before they can reach clinical use [39,43]. Conversely, untargeted Zn-chelating agents, despite their historical role, rank lowest in translational potential due to poor isoform selectivity and systemic toxicity [5,43].

Based on these examples, the approach outlined here can guide the design of antibacterial agents that are structurally sound, effectively delivered, and less likely to cause resistance—helping align metalloprotein-targeted drugs with sustainable and personalized medicine.

## 8. Case Studies of Metalloprotein-Targeted Antibacterial Strategies

**Figure 10:**
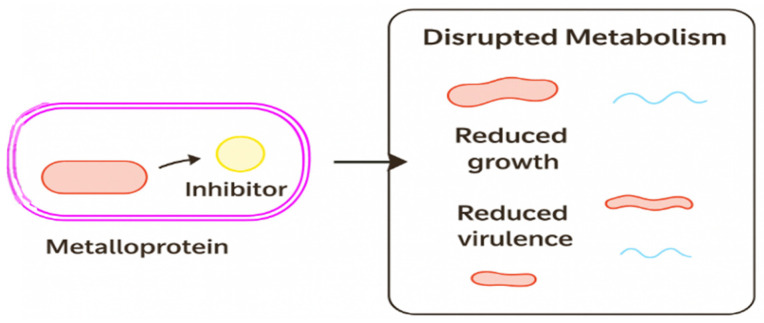
Conceptual impact of metalloprotein inhibition on bacterial physiology. An inhibitor targeting a bacterial metalloprotein disrupts metal-dependent enzymatic pathways, leading to impaired metabolism. This disruption reduces bacterial growth and virulence, highlighting the therapeutic potential of metalloprotein-targeted strategies in antimicrobial development.

Targeting bacterial metalloproteins has led to several therapeutic strategies that exploit distinct aspects of metal homeostasis. Although these approaches differ in their molecular targets, they share common principles related to metal coordination, enzymatic vulnerability, and pathogen-specific uptake mechanisms. A conceptual overview of how metalloprotein inhibition disrupts bacterial physiology is provided (Figure 10).

### 8.1. Iron-Targeting Strategies: Chelation, Mimicry, and Siderophore Hijacking

Iron metabolism is one of the most extensively explored metalloprotein-related targets.

Broad iron chelation using agents such as 2,2′-bipyridyl deprives bacteria of ferric ions required for respiration, DNA synthesis, and enzymatic catalysis. In *Pseudomonas aeruginosa*, iron deprivation reduces biofilm biomass and suppresses quorum-sensing regulators, while in *Escherichia coli* it disrupts siderophore-mediated uptake, leading to bacteriostasis [50,53,56].

Gallium-based therapeutics extend this concept by mimicking Fe^3+^ and competitively inhibiting iron-dependent enzymes. Gallium nitrate has progressed to clinical trials for cystic fibrosis infections, demonstrating translational potential, although its narrow therapeutic window and host toxicity limit broader use [46].

**Figure 11:**
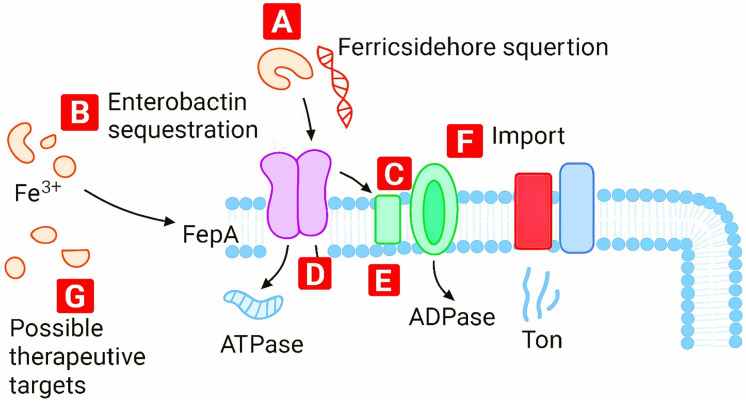
Mechanism of ferric siderophore import and therapeutic intervention points in bacterial cells. (**A**) Ferric siderophore secretion into the extracellular environment initiates the acquisition of iron. (**B**) Enterobactin binds Fe^3+^ with high affinity and is recognized by outer membrane transporter FepA. (**C**) FepA facilitates translocation of the ferric siderophore complex across the outer membrane. (**D**) ATPase and (**E**) ADPase provide energy for transport via the TonB-dependent system. (**F**) The ferric complex is imported into the cytoplasm for metabolic use. (**G**) Potential therapeutic targets include siderophore biosynthesis, transporter inhibition, and disruption of energy coupling.

A more selective approach is siderophore–antibiotic conjugation, which hijacks pathogen-specific Fe^3+^ transporters. Cefiderocol uses catecholate-mediated Fe^3+^ chelation to enter Gram-negative bacteria through TonB-dependent receptors such as FepA and CirA. This strategy achieves higher specificity than gallium or broad chelators but remains vulnerable to transporter variability and bypass pathways [51]. The ferric siderophore import pathway and its intervention points are shown (Figure 11).

Overall, iron-targeting strategies are most successful when they combine high-affinity metal coordination with pathogen-specific uptake mechanisms rather than relying solely on metal deprivation [52].

### 8.2. Zinc-Directed Inhibition of Metallo-β-Lactamases

Zinc sequestration is particularly effective against metallo-β-lactamases (MBLs) such as NDM-1, which require Zn^2+^ for hydrolytic activity. In murine models infected with NDM-1-producing *Klebsiella pneumoniae*, zinc deprivation significantly reduces bacterial burden [51,52]. However, broad Zn-chelators risk off-target toxicity because Zn-dependent enzymes are abundant in host tissues.

To address this limitation, reversible Zn^2+^-coordinating inhibitors such as OP607 have been developed. These compounds bind the catalytic Zn^2+^ ions without displacing them or altering the enzyme’s structure, restoring carbapenem efficacy while minimizing toxicity [35]. This principle—selective inhibition through transient Zn^2+^ coordination—is illustrated conceptually (Figure 10).

Compared with iron-targeting strategies, zinc-directed therapeutics require more precise coordination chemistry to avoid host toxicity, making reversible inhibitors more promising than broad chelators.

### 8.3. Copper-Based Redox Disruption

Copper-based agents exploit redox cycling to generate reactive oxygen species (ROS) within bacterial cells. In *E. coli*, copper complexes overwhelm antioxidant defenses, reduce catalase activity, and trigger oxidative-stress-mediated cell death [35].

Although mechanistically potent, these agents lack pathogen specificity because mammalian cells also rely on redox balance. As a result, controlled delivery platforms are essential to localize copper activity to infection sites and reduce systemic toxicity.

Copper-based strategies therefore require delivery-matched design, unlike siderophore conjugates or Zn-specific inhibitors that inherently possess selectivity.

### 8.4. Emerging Modalities: Metallo-PROTACs and Responsive Nanoparticles

New therapeutic modalities expand the metalloprotein-targeting landscape beyond traditional chelators and enzyme inhibitors.

Metallo-PROTACs irreversibly degrade metalloproteins rather than transiently inhibiting them. Platinum-based PROTACs targeting thioredoxin reductase achieve complete enzyme clearance in resistant *Staphylococcus aureus*, demonstrating catalytic efficiency [57].

Responsive nanoparticles represent another innovation. These systems release inhibitors only in infection sites with elevated protease activity, reducing systemic toxicity and improving therapeutic precision [52]. Their design integrates metal-targeting chemistry with environmental responsiveness.

A systems-level map of the iron-uptake network in Gram-negative bacteria highlights regulatory nodes that govern siderophore biosynthesis, transport, and feedback control (Figure 11). This perspective supports rational design of interventions that disrupt metal trafficking at multiple points.

A structured comparison of metalloprotein-targeting strategies, including their advantages, limitations, and representative examples, is provided (Table 3).

### 8.5. Cross-Cutting Principles and Therapeutic Implications

Across these case studies, several unifying principles emerge:

- Potency vs. selectivity trade-off: Zn-chelators and copper complexes are potent but nonspecific, whereas biologics and non-chelating binders achieve higher selectivity but face delivery challenges.
- Structural knowledge drives success: Strategies such as OP607 require detailed structural data, underscoring the importance of crystallography and SAR-guided optimization.
- Delivery determines therapeutic index: Siderophore conjugates and responsive nanoparticles outperform free chelators by ensuring pathogen-specific uptake and minimizing host exposure.
- Mechanistic durability: Metallo-PROTACs offer irreversible inhibition, while reversible inhibitors provide tunable, safer activity.

Collectively, these insights show that metalloprotein-targeted antibacterial strategies are most effective when they:

1. Exploit rigid or highly conserved metal-binding architectures.
2. Leverage pathogen-specific uptake pathways.
3. Incorporate delivery platforms that enhance selectivity.
4. Align with systems-level understanding of metal trafficking.

By integrating structural biology, bioinorganic chemistry, and infection pharmacology, metal-focused therapeutics can progress from conceptual promise to clinically actionable interventions [52,55].

While multiple studies support the efficacy of gallium-based antimicrobials in disrupting iron metabolism, their translational potential remains limited by host toxicity and narrow-spectrum activity. In contrast, manganese chelation strategies appear more promising due to their broader impact on oxidative-stress pathways, although the risk of off-target effects on host metalloproteins must be carefully evaluated. These observations suggest that dual-targeting approaches, such as combining metal chelation with efflux-pump inhibition, may offer a more robust therapeutic window [42]. These case studies validate the mechanistic rationale behind metalloprotein targeting while emphasizing the need for pathogen-specific selectivity, scalable delivery platforms, and regulatory frameworks attuned to next-generation antimicrobial agents.

## 9. Strategic Challenges and Translational Pathways in Metalloprotein Targeting

Even though we have learned a lot about how bacterial metalloproteins work and how they could be targeted by new drugs, research in this area is still at an early stage. Only a limited number of bacterial metalloproteins have been characterized in sufficient detail, and the biochemistry and structure–activity relationships of their inhibitors are still poorly understood. As drug design moves into less familiar areas of chemistry, it needs both careful testing and creative thinking.

One key challenge is the limited success of target-based computational methods, such as molecular docking and computer-aided drug design (CADD), in developing effective inhibitors for bacterial metalloproteins like metallothioneins. Although target-based screening against many metalloprotein families has yielded promising enzyme inhibitors, these often fail in cellular systems where the targeted proteins can be circumvented through redundant or compensatory pathways [58]. To deal with this complexity, we will need approaches that balance broad antibacterial effects with enough precision to avoid resistance through alternate pathways. For example, TEM-1 β-lactamase, despite a 50% invariant sequence, can develop resistance through mutations affecting only ~10% of its active site residues, dramatically altering acylation rates and antibiotic hydrolysis [59]. To design inhibitors that stay effective, researchers need to understand and anticipate possible mutations using structural, genomic, and evolutionary data [60].

Another major challenge is making sure these drugs are selective enough to hit the right bacterial proteins without affecting similar ones in humans. Target proteins often exist as multiple isoforms with over 90% sequence similarity to non-target homologs, which can lead to off-target effects. Researchers are now using advanced structural and computational tools to predict which parts of proteins bind metals, find possible off-target effects, and fine-tune drug molecules for better selectivity [61]. Integrative bioinformatics pipelines that match three-dimensional protein models with selective ligands enable rational design of compounds with controlled metal coordination geometries and predictable binding affinities [62]. This approach, when coupled with experimental validation of metal–ligand complexes, represents a promising pathway toward achieving selectivity without compromising potency [63].

Beyond selectivity, regulatory and developmental hurdles remain formidable. Despite the urgent global demand for new antimicrobials, the clinical pipeline remains alarmingly sparse. Only a handful of novel antibiotics have reached late-stage trials, and most are derivatives of existing scaffolds [64]. Encouragingly, pharmacological screenings of hundreds of metal-containing compounds have revealed dozens of potent activities against both Gram-positive and Gram-negative strains, demonstrating the untapped potential of metal-based complexes [65].

Gallium, a Group IIIA metal, is structurally and chemically like ferric iron (Fe^3+^), allowing it to mimic iron and interfere with bacterial iron metabolism. Unlike iron, gallium is redox-inactive and cannot participate in Fenton reactions or catalyze essential enzymatic processes. This unique property enables gallium compounds to disrupt iron-dependent pathways in bacteria, leading to impaired DNA synthesis, respiration, and oxidative stress management. Multiple in vitro and in vivo studies have demonstrated the antimicrobial activity of gallium-based compounds, particularly against *Pseudomonas aeruginosa* and other iron-scavenging pathogens. Gallium nitrate and gallium-protoporphyrin IX have shown efficacy in inhibiting biofilm formation and growth by targeting iron-regulated virulence systems. These findings underscore gallium’s potential as an iron mimetic therapeutic agent. However, their translational application remains constrained by host toxicity at therapeutic concentrations and a relatively narrow antimicrobial spectrum [66]. Additionally, delivery and stability challenges in physiological environments further limit clinical advancement.

To overcome these barriers, future research must adopt a holistic, genome-to-phenotype approach. Although progress in metalloprotein biology has been significant, much of our knowledge is derived from a small subset of model organisms such as *E. coli* or *S. aureus* [23]. Predicting metalloproteins at a genomic scale, across diverse pathogenic taxa, is essential for mapping their evolutionary distribution, functional roles, and virulence associations. Comparative genomics and proteomics now enable the mining of entire microbial lineages for conserved and divergent metalloprotein families, offering a foundation for precision-targeted drug discovery. Integrating computational predictions with biochemical assays, structural biology, and cellular validation can transform these genomic insights into actionable therapeutic leads.

Equally critical are collaborative research initiatives and global capacity building. Addressing the escalating crisis of antibiotic resistance demands sustained international cooperation, encompassing not only data sharing and technology transfer but also long-term training and infrastructure development. Collaborative networks that unite computational biologists, chemists, and microbiologists are essential for translating fundamental discoveries into viable therapeutics. Educational initiatives must therefore expand to include interdisciplinary training in bioinorganic chemistry, computational biology, and translational microbiology, ensuring the next generation of scientists can tackle these complex challenges effectively.

At the global health level, antibiotic resistance represents one of the most pressing biomedical threats of the 21st century. Effective mitigation requires cross-border collaboration, particularly in regions where resistant infections are most prevalent, Africa, the Indian subcontinent, and South America. Establishing new laboratories, promoting access to diagnostic technologies, and fostering research partnerships in these regions are crucial for a coordinated response. Moreover, the rise of multidrug-resistant Gram-negative bacteria poses unique challenges due to their impermeable outer membranes, diverse virulence factors, and rapid horizontal gene transfer. Innovative approaches such as microbiome engineering, synthetic microbial competition, and bio-inspired drug delivery systems offer new hope for counteracting these resilient pathogens.

In parallel, ethical and regulatory frameworks must evolve to ensure that antimicrobial research complies with biosafety and responsible research guidelines. According to European Commission (EC) directives on BSL-2 organisms, all research involving pathogenic bacteria must adhere to established Risk Assessment Protocols and Responsible Research and Innovation (RRI) standards [23,66].

Collaborative projects that integrate ethics training, open decision-making, and transparent data management represent a model for sustainable, ethically aligned scientific progress.

## 10. Limitations of This Review

While this review integrates extensive literature, several limitations exist:

- Scope of Literature Coverage: Although this review integrates evidence from major databases (PubMed, Scopus, Web of Science), it may not capture all emerging studies published after the search window or in non-indexed journals. As the field of metallodrug chemistry and nanoparticle design continues to advance rapidly, some recent results may not yet be included here. Rapid developments in metallodrug chemistry and nanoparticle engineering may introduce new findings not reflected in this overview.
- Variability in Experimental Methodologies: The studies summarized employ diverse experimental systems, ranging from in vitro biochemical assays to in vivo infection models, which complicates direct comparison. Variations in metal levels, experimental setups, or how closely tests mimic real infection conditions can affect how effective an inhibitor appears to be.
- Limited Clinical Translation Data: Many metalloprotein-targeted strategies remain in preclinical stages. As a result, conclusions regarding therapeutic potential are based primarily on mechanistic and early-stage experimental evidence rather than large-scale clinical validation.
- Pathogen-Specific Differences: Metalloprotein function and metal-homeostasis pathways vary across bacterial species. While this review highlights generalizable principles, some mechanistic insights may not apply uniformly to all pathogens, particularly those with unique metal-acquisition systems or regulatory networks.
- Structural Data Gaps: For several metalloproteins of therapeutic interest, high-resolution structural information remains incomplete. This limits the precision of mechanism-informed inhibitor design and may bias the review toward better-characterized targets such as metallo-β-lactamases and siderophore receptors.

Being aware of these limitations highlights the necessity for increased collaboration among structural biologists, microbiologists, chemists, and pharmacologists to advance this research. Recognizing these limitations underscores the need for ongoing research. Addressing these gaps is essential for advancing metalloprotein-targeted therapeutics.

## 11. Concluding Perspectives

Metalloproteins play a central role in how bacteria function. They help manage oxidative stress, take up nutrients, repair DNA, and adjust to changes in metal availability caused by the host. By integrating structural biology, coordination chemistry, and mechanistic enzymology, this review highlights how these metal-dependent systems create exploitable vulnerabilities for antibacterial development. This review shows how understanding the chemistry and structure of these metal-dependent systems can guide the development of new antibacterial drugs. It also offers a way to decide which metalloproteins are worth targeting, depending on how rigid their metal-binding sites are and how well they can adapt to change.

Across the therapeutic modalities examined, small-molecule inhibitors, biologics, and catalytic metallodrugs, several cross-cutting principles emerge. Across all drug types, small molecules, biologics, and catalytic metallodrugs, a few key themes repeat. The most successful approaches fit the drug’s structure to the shape of the metal site, take advantage of metal-driven redox reactions, or block the metal transport systems bacteria depend on to survive infection. At the same time, shared bottlenecks such as active-site remodeling, competition with host metal-binding proteins, and redox-dependent conformational shifts underscore the need for metal-aware infection models and mechanistic validation under physiologically relevant conditions.

At the same time, challenges remain. Bacteria can alter their active sites, compete with host proteins for metal ions, or change shape in response to redox conditions. To address this, infection models need to reflect real metal conditions inside the body. New tools, such as cryo-electron microscopy, structure prediction, and metal analysis, will make it easier to identify suitable targets and design more effective inhibitors. Connecting these methods with our understanding of how bacteria handle metals could help transform these ideas into safer, more effective treatments. Designing drugs that follow the actual molecular logic of metal-dependent biology may also make them less toxic and more resistant to bacterial adaptation. Designing antibacterial agents based on the molecular principles of metal-dependent biology may improve selectivity and reduce toxicity. Such strategies may also improve therapeutic durability against emerging resistance.

## Figures and Tables

**Table 1:** Selective bacterial metalloprotein inhibitors and their molecular mechanisms of action. This table summarizes representative inhibitors targeting bacterial metalloproteins, detailing their mechanisms of action, delivery strategies, selectivity indices, toxicity profiles, and stages of clinical development. To enhance molecular insight, each entry highlights the underlying coordination chemistry, metal-binding mode, and structural features that contribute to inhibitor potency and selectivity. All inhibitors were identified using the systematic literature-screening approach described in the Introduction.

Metal Coordination and Structural Details	Clinical Stage	Toxicity	Selectivity	Delivery	Target Mechanism	Agent
N,N-bidentate ligation; octahedral Fe^3+^; PDB: n/a	Preclinical	Potential host metal depletion	Moderate	Systemic	Chelates Fe^3+^, disrupts metalloenzymes	2,2′-Bipyridyl [1,14]
Hexadentate catecholate; stable octahedral Fe^3+^; PDB: 1FEP	Experimental (in vivo)	Low	High	Bacterial secretion	Fe^3+^ sequestration	Enterobactin [1,4]
Dinuclear Zn^2+^ center; His/Asp/Cys ligands; PDB: 3SPU, 4EYB	Phase I/II	Minimal off-target effects	High	IV/oral	Zinc-binding β-lactamase	NDM-1 Inhibitors [2]
O,O-donor ligands; octahedral Fe; PDB: n/a	Preclinical	Low	High	Topical/systemic	Chelates Fe^3+^, inhibits biofilm	OP607 [12,13]
Extracellular His/Cys motifs; geometry target-dependent; PDB varies	Early	Low	Very high	IV	Metalloprotein neutralization	Antibody inhibitors [27]

**Table 2:** Key bacterial metalloprotein targets, their virulence-associated functions, and corresponding therapeutic strategies. This table provides an overview of major bacterial metalloproteins implicated in pathogenicity, including their associated pathogens, molecular mechanisms, and roles in metal-dependent virulence pathways. For each target class, the table outlines therapeutic approaches, supporting experimental evidence, mechanistic advantages, and known limitations. Entries were compiled using the literature identification strategy described in the Introduction, with an emphasis on structural and mechanistic features relevant to antibacterial tractability.

Limitations	Advantages	Evidence	Therapeutic Strategy	Mechanism	Pathogens	Target Class
Selectivity challenges	Unique bacterial targets	In vitro inhibition	Small molecule inhibitors	Copper uptake and oxidative stress (Cu^+^/Cu^2+^ via Met-/His-rich motifs; linear/trigonal planar; PDB: CopA 3RFU)	*P. aeruginosa*	Copper Transport Proteins [32]
Resistance via transporter mutations	Pathogen-specific	Mouse peritonitis, UTI models	Siderophore analogs, chelators	High-affinity Fe^3+^ acquisition (Fe^3+^ octahedral; catecholate/hydroxamate; FepA/BauA; PDB: 1FEP,5FP1)	*E. coli*	Iron-Siderophore Systems [1,4]
Resistance via transporter mutations	Pathogen-specific	Mouse peritonitis, UTI models	Siderophore analogs, chelators	High-affinity Fe^3+^ acquisition (Fe^3+^ octahedral; catecholate/hydroxamate; FepA/BauA; PDB: 1FEP, 5FP1)	*S. aureus*	
Risk of off-target ROS imbalance	Direct virulence target	Genetic knockout and virulence models	Enzyme inhibitors	ROS detoxification (Mn^2+^ trigonal bipyramidal; His26/His81/Asp167/His171; PDB: 2XDA)	*S. aureus*	Mn-Superoxide Dismutase [2]
Host enzyme similarity	Essential enzymes	Structural biology + inhibitor screens	Zn-binding site inhibitors	DNA replication/repair (Zn^2+^ tetrahedral; His/Cys/Asp residues; PDB: 3A1J)	*N. gonorrhoeae*	Zn-dependent Enzymes [3]
Redundancy across strains	Virulence disruption	In vitro and tissue models	Protease inhibitors	Host tissue invasion (Zn^2+^ catalytic center; His-Glu-His triad; tetrahedral; PDB: 1QJX)	*H. pylori*	Metalloproteinases [33]

**Table 3:** Comparative overview of metalloprotein-targeting strategies. This table summarizes major therapeutic approaches used to modulate metalloprotein activity, comparing zinc-chelating agents, non-chelating binders, peptides, biologics, catalytic metallodrugs, targeted degraders, and responsive delivery systems. Strategies are evaluated based on potency, specificity, and mechanistic limitations. Abbreviations: MMP, matrix metalloproteinase; SAR, structure–activity relationship; ADAMTS, a disintegrin and metalloproteinase with thrombospondin motifs; TIMP, tissue inhibitor of metalloproteinases; ATCUN, amino-terminal copper and nickel binding motif; PROTAC, proteolysis-targeting chimera; Trx1/TrxR1, thioredoxin 1/thioredoxin reductase 1.

Representative Example	Disadvantages	Advantages	Strategy Type
Broad-spectrum MMP inhibitors (e.g., batimastat, marimastat)	Poor isoform selectivity; systemic toxicity (e.g., musculoskeletal pain)	High potency; minimal structural complexity	Zn-chelating small molecules [5,43]
S1′-pocket-targeted MMP-7 inhibitors	Requires detailed structural data; extensive SAR optimization	Isoform selectivity; preserves native metal coordination	Non-chelating pocket binders [7,8]
ADAMTS-selective peptide inhibitors	Proteolytic instability; delivery and bioavailability challenges	High affinity; modular and tunable design	Peptides/Peptidomimetics [16]
Engineered TIMPs	High production cost; limited tissue penetration; potential immunogenicity	High specificity; long circulating half-life	Biologics (TIMPs, antibodies) [25,27]
ATCUN-motif-based metallodrugs	Metal lability; risk of systemic toxicity	Novel catalytic mechanisms; prodrug activation potential	Catalytic metallodrugs [18,43]
Pt-PROTACs degrading Trx1/TrxR1	Large molecular size; permeability and E3-ligase recruitment constraints	Permanent target removal; catalytic mode of action	Targeted degraders (metallo-PROTACs) [43]
MMP-responsive nanoparticles	Complex formulation; variable enzyme expression across tissues	Spatial control; reduced systemic exposure	Responsive delivery systems [13]

## Data Availability

No new data were created or analyzed in this study. Data sharing is not applicable to this article.

## References

[1] Neto J.F.d.S., Staats C.C., Pontes M.H. (2023). Editorial: Metal homeostasis in microbial physiology and virulence. Front. Cell. Infect. Microbiol..

[2] Begg S.L. (2019). The role of metal ions in the virulence and viability of bacterial pathogens. Biochem. Soc. Trans..

[3] Choi Y., Koh J., Cha S., Roe J. (2024). Activation of zinc uptake regulator by zinc binding to three regulatory sites. Nucleic Acids Res..

[4] Chandrangsu P., Rensing C., Helmann J.D. (2017). Metal homeostasis and resistance in bacteria. Nat. Rev. Microbiol..

[5] Karnwal A., Jassim A.Y., Mohammed A.A., Said A.R.M., Selvaraj M., Malik T. (2025). Addressing the global challenge of bacterial drug resistance: Insights, strategies, and future directions. Front. Microbiol..

[6] Vitali V., Zineddu S., Messori L. (2025). Metal compounds as antimicrobial agents: Smart approaches for discovering new effective treatments. RSC Adv..

[7] Simmons B. (2010). Clinical reasoning: Concept analysis. J. Adv. Nurs..

[8] Kehl-Fie T.E., Skaar E.P. (2010). Nutritional immunity beyond iron: A role for manganese and zinc. Curr. Opin. Chem. Biol..

[9] Shirakawa K.T., Sala F.A., Miyachiro M.M., Job V., Trindade D.M., Dessen A. (2023). Architecture and genomic arrangement of the MurE–MurF bacterial cell wall biosynthesis complex. Proc. Natl. Acad. Sci. USA.

[10] Cassat J.E., Skaar E.P. (2013). Iron in infection and immunity. Cell Host Microbe.

[11] Hood M.I., Skaar E.P. (2012). Nutritional immunity: Transition metals at the pathogen–host interface. Nat. Rev. Microbiol..

[12] Rossolini G.M. (2015). Extensively drug-resistant carbapenemase-producing Enterobacteriaceae. J. Intern. Med..

[13] King A.M., Reid-Yu S.A., Wang W., King D.T., De Pascale G., Strynadka N.C., Walsh T.R., Coombes B.K., Wright G.D. (2014). AMA overcomes antibiotic resistance by NDM and VIM metallo-β-lactamases. Nature.

[14] Palica K., Deufel F., Skagseth S., Di Santo Metzler G.P., Thoma J., Andersson Rasmussen A., Valkonen A., Sunnerhagen P., Leiros H.-K.S., Andersson H. (2023). α-Aminophosphonate inhibitors of metallo-β-lactamases NDM-1 and VIM-2. RSC Med. Chem..

[15] Hijazi A.K., El-Khateeb M., Taha Z.A., Alomari M.I., Khwaileh N.M., Alakhras A.I., Al-Momani W.M., Elrashidi A., Barham A.S. (2025). Anti-Bacterial and Anti-Fungal Properties of a Set of Transition Metal Complexes Bearing a Pyridine Moiety and [B(C_6_F_5_)_4_]_2_ as a Counter Anion. Molecules.

[16] Beck A., Goetsch L., Dumontet C., Corvaïa N. (2017). Strategies and challenges for the next generation of antibody–drug conjugates. Nat. Rev. Drug Discov..

[17] Liu F., Su Z., Chen P., Tian X., Wu L., Tang D., Li P., Deng H., Ding P., Fu Q. (2021). Structural basis for zinc-induced activation of a zinc uptake transcriptional regulator. Nucleic Acids Res..

[18] Šink R., Kotnik M., Zega A., Barreteau H., Gobec S., Blanot D., Dessen A., Contreras-Martel C. (2016). Crystallographic Study of Peptidoglycan Biosynthesis Enzyme MurD: Domain Movement Revisited. PLoS ONE.

[19] Antimicrobial Resistance Collaborators (2022). Global burden of bacterial AMR in 2019. Lancet.

[20] Huang Z., Bian X., Li Y., Hu J., Guo B., Yang X., Jin Y., Zheng S., Wang X., Gao C. (2024). In vitro pharmacokinetics/pharmacodynamics of FL058 (a novel β-lactamase inhibitor) combined with meropenem against carbapenemase-producing Enterobacterales. Front. Pharmacol..

[21] Miethke M., Pieroni M., Weber T., Brönstrup M., Hammann P., Halby L., Arimondo P.B., Glaser P., Aigle B., Bode H.B. (2021). Towards the sustainable discovery and development of new antibiotics. Nat. Rev. Chem..

[22] Porcheron G., Garenaux A., Proulx J., Sabri M., Dozois C.M. (2013). Iron, copper, zinc, and manganese transport and regulation in pathogenic *Escherichia coli*. Front. Cell. Infect. Microbiol..

[23] Wang Y., Wang S., Chen W., Song L., Zhang Y., Shen Z., Yu F., Li M., Ji Q. (2018). CRISPR-Cas9 and CRISPR-Assisted Cytidine Deaminase Enable Precise and Efficient Genome Editing in Klebsiella pneumoniae. Appl. Environ. Microbiol..

[24] Song Y., Wu X., Li Z., Ma Q.Q., Bao R. (2024). Molecular mechanism of siderophore regulation by the Pseudomonas aeruginosa BfmRS two-component system in response to osmotic stress. Commun. Biol..

[25] Capdevila D.A., Wang J., Giedroc D.P. (2016). Zinc metallostasis at the host–pathogen interface. J. Biol. Chem..

[26] Djoko K.Y., Ong C.L., Walker M.J., McEwan A.G. (2015). Copper and zinc toxicity in innate immunity. J. Biol. Chem..

[27] Kumar L., Bisen M., Harjai K., Chhibber S., Azizov S., Lalhlenmawia H., Kumar D. (2023). Advances in Nanotechnology for Biofilm Inhibition. ACS Omega.

[28] Lemire J., Harrison J., Turner R. (2013). Antimicrobial activity of metals: Mechanisms, molecular targets and applications. Nat. Rev. Microbiol..

[29] Jiang D., Ye Z., Hsieh C.-Y., Yang Z., Zhang X., Kang Y., Du H., Wu Z., Wang J., Zeng Y. (2023). MetalProGNet: A structure-based deep graph model for metalloprotein–ligand interaction predictions. Chem. Sci..

[30] Jumper J., Evans R., Pritzel A., Green T., Figurnov M., Ronneberger O., Tunyasuvunakool K., Bates R., Žídek A., Potapenko A. (2021). Highly accurate protein structure prediction with AlphaFold. Nature.

[31] Cheng Y. (2015). Single-particle cryo-EM at crystallographic resolution. Cell.

[32] Costa S., Ragusa M.A., Buglio G.L., Scilabra S.D., Nicosia A. (2022). The Repertoire of Tissue Inhibitors of Metalloproteases: Evolution, Regulation of Extracellular Matrix Proteolysis, Engineering and Therapeutic Challenges. Life.

[33] Palmer L.D., Skaar E.P. (2016). Transition metals and virulence. Annu. Rev. Genet..

[34] Kim J.J., Kim Y.S., Kumar V. (2019). Heavy metal toxicity: An update of chelating therapeutic strategies. J. Trace Elem. Med. Biol..

[35] Zhang H., Hao Q. (2011). Crystal structure of NDM-1 reveals a common β-lactam hydrolysis mechanism. FASEB J..

[36] Jain R.K., Stylianopoulos T. (2010). Delivering nanomedicine to solid tumors. Nat. Rev. Clin. Oncol..

[37] Békés M., Langley D.R., Crews C.M. (2022). PROTAC targeted protein degraders: The past is prologue. Nat. Rev. Drug Discov..

[38] Su J., Li Z., Liao B., Zhu Y., Zhang X., Wang C., He J. (2017). Microcalorimetric study of the effect of manganese on the growth and metabolism in a heterogeneously expressing manganese-dependent superoxide dismutase (Mn-SOD) strain. J. Therm. Anal. Calorim..

[39] Gasparrini A.J., Wang B., Sun X., Kennedy E.A., Hernandez-Leyva A., Ndao I.M., Tarr P.I., Warner B.B., Dantas G. (2019). Persistent metagenomic signatures of early-life hospitalization and antibiotic treatment in the infant gut microbiota and resistome. Nat. Microbiol..

[40] Lysenko V., Gao M.-L., Sterk F.A.C., Innocenti P., Slingerland C.J., Martin N.I. (2025). Design, Synthesis, and Antibacterial Evaluation of Rifampicin–Siderophore Conjugates. ACS Infect. Dis..

[41] Krokidis M.G., Koumadorakis D.E., Lazaros K., Ivantsik O., Exarchos T.P., Vrahatis A.G., Kotsiantis S., Vlamos P. (2025). AlphaFold3: An Overview of Applications and Performance Insights. Int. J. Mol. Sci..

[42] Becker K.W., Skaar E.P. (2014). Metal limitation and toxicity at the interface between host and pathogen. FEMS Microbiol. Rev..

[43] Poole K. (2017). At the nexus of antibiotics and metals: The impact of Cu and Zn on antibiotic activity and resistance. Trends Microbiol..

[44] Sherry N.L., Lee J.Y.H., Giulieri S.G., Connor C.H., Horan K., Lacey J.A., Lane C.R., Carter G.P., Seemann T., Egli A. (2025). Genomics for antimicrobial resistance—Progress and future directions. Antimicrob. Agents Chemother..

[45] Kasmanas J.C., Magnúsdóttir S., Zhang J., Smalla K., Schloter M., Stadler P.F., Ponce A.C., Rocha U. (2025). Integrating comparative genomics and risk classification by assessing virulence, antimicrobial resistance, and plasmid spread in microbial communities with gSpreadComp. GigaScience.

[46] Olsen N.S., Riber L. (2025). Metagenomics as a transformative tool for antibiotic resistance surveillance: The role of mobile genetic elements and phages. Antibiotics.

[47] Gräff Á.T., Barry S.M. (2024). Siderophores as tools and treatments. npj Antimicrob. Resist..

[48] Garau G., Di Guilmi A.M., Hall B.G. (2005). Structure-based phylogeny of the metallo-β-lactamases. Antimicrob. Agents Chemother..

[49] Haloi N., Howard R.J., Lindahl E. (2025). Cryo-EM ligand building using an AlphaFold3-like model and molecular dynamics. PLoS Comput. Biol..

[50] Keskey R.C., Xiao J., Hyoju S., Lam A., Kim D., Sidebottom A.M., Zaborin A., Dijkstra A., Meltzer R., Thakur A. (2025). Enterobactin inhibits microbiota-dependent activation of AhR to promote bacterial sepsis in mice. Nat. Microbiol..

[51] Katsube T., Echols R., Arjona Ferreira J.C., Krenz H.K., Berg J.K., Galloway C. (2017). Cefiderocol, a siderophore cephalosporin for Gram-negative bacterial infections: Pharmacokinetics and safety in subjects with renal impairment. J. Clin. Pharmacol..

[52] Sheldon J.R., Skaar E.P. (2020). *Acinetobacter baumannii* can use multiple siderophores for iron acquisition, but only acinetobactin is required for virulence. PLoS Pathog..

[53] Francine P. (2022). Systems biology: New insight into antibiotic resistance. Microorganisms.

[54] Liu Y., Xu Y., Xu X., Chen X., Chen H., Zhang J., Ma J., Zhang W., Zhang R., Chen J. (2023). Metagenomic identification of pathogens and antimicrobial-resistant genes in bacterial positive blood cultures by nanopore sequencing. Front. Cell. Infect. Microbiol..

[55] Colaço H.G., Santo P.E., Matias P.M., Bandeiras T.M., Vicente J.B. (2016). Roles of *Escherichia coli* ZinT in cobalt, mercury and cadmium resistance and structural insights into the metal-binding mechanism. Metallomics.

[56] Schalk I.J. (2025). Bacterial siderophores: Diversity, uptake pathways and applications. Nat. Rev. Microbiol..

[57] Serebnitskiy Z., Orban K., Finkel S.E. (2025). A role for Dps ferritin activity in long-term survival of *Escherichia coli*. Microbiol. Spectr..

[58] Zhu C., Diao Z., Yang Y., Liao J., Wang C., Li Y., Liang Z., Xu P., Liu X., Zhang Q. (2025). Recent advances and challenges in metal-based antimicrobial materials: A review of strategies to combat antibiotic resistance. J. Nanobiotechnol..

[59] Sandy M., Butler A. (2009). Microbial iron acquisition: Marine and terrestrial siderophores. Chem. Rev..

[60] Alhayek A., Hirsch A.K.H. (2023). Bacterial metalloproteases as promising drug targets for antivirulence agents. Annu. Rep. Med. Chem..

[61] Gasser G., Metzler-Nolte N. (2012). The potential of organometallic complexes in medicinal chemistry. Curr. Opin. Chem. Biol..

[62] Wong D., Nielsen T.B., Bonomo R.A., Pantapalangkoor P., Luna B., Spellberg B. (2017). Clinical and pathophysiological overview of *Acinetobacter* infections: A century of challenges. Clin. Microbiol. Rev..

[63] Almeida M.R., Videira M.A., Lima J.C., Saraiva L. (2025). Mechanistic insights into *Staphylococcus aureus* IsdG-ferrochelatase interactions: A key to understanding haem homeostasis in pathogens. J. Inorg. Biochem..

[64] Frei A., Zuegg J., Elliott A.G., Baker M., Bräse S., Brown C., Chen F., Dowson C.G., Dujardin G., Jung N. (2020). Metal complexes as a promising source for new antibiotics. Chem. Sci..

[65] Kayode M., Ayodeji O.E., Yetunde B.B., Joseph O.T., Timothy A.T., Habeeb O., Tochi N.S., Alice A.A., Onyemeh O., Okeowo H. (2024). Synthesis, characterization and antimicrobial activity of mixed antibiotic–vitamin metal complexes involving metronidazole and thiamine. Asian J. Chem. Sci..

[66] Nahar L., Hagiya H., Gotoh K., Asaduzzaman M., Otsuka F. (2024). New Delhi metallo-β-lactamase inhibitors: A systematic scoping review. J. Clin. Med..

